# Mining for novel cyclomaltodextrin glucanotransferases unravels the carbohydrate metabolism pathway via cyclodextrins in Thermoanaerobacterales

**DOI:** 10.1038/s41598-021-04569-x

**Published:** 2022-01-14

**Authors:** Sara Centeno-Leija, Laura Espinosa-Barrera, Beatriz Velazquez-Cruz, Yair Cárdenas-Conejo, Raúl Virgen-Ortíz, Georgina Valencia-Cruz, Roberto A. Saenz, Yerli Marín-Tovar, Saúl Gómez-Manzo, Beatriz Hernández-Ochoa, Luz María Rocha-Ramirez, Rocío Zataraín-Palacios, Juan A. Osuna-Castro, Agustín López-Munguía, Hugo Serrano-Posada

**Affiliations:** 1 Consejo Nacional de Ciencia y Tecnología, Laboratorio de Biología Sintética, Estructural y Molecular, Laboratorio de Agrobiotecnología, Tecnoparque CLQ, Universidad de Colima, Carretera Los Limones-Loma de Juárez, 28627 Colima, Colima Mexico; 2 Laboratorio de Biología Sintética, Estructural y Molecular, Laboratorio de Agrobiotecnología, Tecnoparque CLQ, Universidad de Colima, Carretera Los Limones-Loma de Juárez, 28627 Colima, Colima Mexico; 3 Centro Universitario de Investigaciones Biomédicas, Universidad de Colima, Avenida 25 de julio 965, Colonia Villa de San Sebastián, 28045 Colima, Colima Mexico; 4 Facultad de Ciencias, Universidad de Colima, Bernal Díaz del Castillo 340, 28045 Colima, Colima Mexico; 5 Laboratorio de Bioquímica Estructural, Departamento de Medicina Molecular y Bioprocesos, Instituto de Biotecnología, Universidad Nacional Autónoma de México, Avenida Universidad 2001, Colonia Chamilpa, 62210 Cuernavaca, Mexico; 6 Laboratorio de Bioquímica Genética, Instituto Nacional de Pediatría, Secretaría de Salud, 04530 Mexico City, Mexico; 7 Laboratorio de Inmunoquímica y Biología Celular, Hospital Infantil de México Federico Gómez, Secretaría de Salud, 06720 Mexico City, Mexico; 8 Unidad de Investigación en Enfermedades Infecciosas, Hospital Infantil de México Federico Gómez, Dr. Márquez No. 162, Colonia Doctores, 06720 Delegación Cuauhtémoc, Mexico; 9 Escuela de Medicina General, Universidad José Martí, Bosques del Decán 351, 28089 Colima, Colima México; 10 Facultad de Ciencias Biológicas y Agropecuarias, Universidad de Colima, Autopista Colima-Manzanillo, 28100 Tecomán, Colima Mexico; 11 Instituto de Biotecnología, Universidad Nacional Autónoma de México, Avenida Universidad 2001, Colonia Chamilpa, 62210 Cuernavaca, Morelos Mexico

**Keywords:** Biochemistry, Microbiology

## Abstract

Carbohydrate metabolism via cyclodextrins (CM-CD) is an uncommon starch-converting pathway that thoroughly depends on extracellular cyclomaltodextrin glucanotransferases (CGTases) to transform the surrounding starch substrate to α-(1,4)-linked oligosaccharides and cyclodextrins (CDs). The CM-CD pathway has emerged as a convenient microbial adaptation to thrive under extreme temperatures, as CDs are functional amphipathic toroids with higher heat-resistant values than linear dextrins. Nevertheless, although the CM-CD pathway has been described in a few mesophilic bacteria and archaea, it remains obscure in extremely thermophilic prokaryotes (T_opt_ ≥ 70 °C). Here, a new monophyletic group of CGTases with an exceptional three-domain ABC architecture was detected by (meta)genome mining of extremely thermophilic Thermoanaerobacterales living in a wide variety of hot starch-poor environments on Earth. Functional studies of a representative member, CldA, showed a maximum activity in a thermoacidophilic range (pH 4.0 and 80 °C) with remarkable product diversification that yielded a mixture of α:β:γ-CDs (34:62:4) from soluble starch, as well as G3–G7 linear dextrins and fermentable sugars as the primary products. Together, comparative genomics and predictive functional analysis, combined with data of the functionally characterized key proteins of the gene clusters encoding CGTases, revealed the CM-CD pathway in Thermoanaerobacterales and showed that it is involved in the synthesis, transportation, degradation, and metabolic assimilation of CDs.

## Introduction

Cyclodextrins (CDs) are cyclic α-(1,4)-linked oligosaccharides that commonly consist of six (α-CD), seven (β-CD) and eight (γ-CD) glucopyranose units, forming a unique truncated cone structure with a hydrophobic central cavity and a hydrophilic outer surface^1^. The CD structure enables the formation of CD-guest complexes through the reversible binding of nonpolar guest molecules (*e.g.*, organic, inorganic, or biological molecules) to increase their solubility, stability, and bioavailability^2^. Since functional characterizations of CDs have been primarily addressed for biotechnological and pharmaceutical applications^3–5^, their physiological purpose has not been thoroughly discussed. Nevertheless, the role of CDs seems to be related to resource competition in microbial communities, such as monopolizing substrate availability or mitigating the toxicity of surrounding organic substrates and volatiles^6,7^, as well as carrying antimicrobial and signaling molecules^8,9^. CDs also act as surfactants by increasing the bioavailability of hydrocarbons in microbial communities living in oil reservoirs^10^. Furthermore, because the glass transition temperature (Tg) of CDs is higher than monosaccharides and linear dextrins^11,12^, starch conversion to CDs is particularly valuable for survival in high-temperature environments.

CDs are synthesized by enzymatic conversion using cyclomaltodextrin glucanotransferases (CGTases; EC 2.4.1.19) through an intramolecular transglycosylation of glucosyl intermediates using starch as substrate (cyclization activity)^13^. CGTases are enzymes that belong to subfamily 2 of the glycoside hydrolase family 13 (GH13_2)^14^. GH13 family (maltodextrin/starch-active enzymes) is the second-largest family of glycoside hydrolases and the principal group of enzymes related to α-amylases, encompassing 44 subfamilies^14,15^. GH13 family belongs to the clan GH-H along with GH70 (sucrose/starch/maltooligosaccharides-active enzymes) and GH77 (amylomaltases) families. All members of the clan GH-H display a catalytic TIM-barrel topology with at least four conserved sequence regions (CSR) from I to IV^16,17^ and display an α-retaining double displacement catalytic mechanism^18^. The four CSR I-IV motifs of the GH13 family contain functionally important residues conserved among CGTases, including an acidic catalytic triad Asp^x^/Glu^y^/Asp^z^, as well as an Arg residue located on the second position before the catalytic nucleophile Asp^x^^16,19^. Moreover, three additional CSR V-VII motifs contain several residues that distinguish the specificities of CGTases from those of other GH13 members^19^. Similar to α-amylases, CGTases can also produce linear oligosaccharides through hydrolysis, disproportionation, or coupling activities^20^. According to the Carbohydrate-Active enZymes (CAZy; http://www.CAZy.org) database^14^, GH13_2 contains 51 characterized enzymes of known sequence that have been isolated from bacteria and archaea, where ~ 80% belong to the well-characterized Gram-positive (G+) mesophilic Bacilli class bacteria, which are distinguished by synthesizing CDs as the primary catalytic product^13^. The overall CGTase fold comprises a multidomain architecture of five domains (ABCDE; ~ 700 residues in total), where domain A adopts a TIM-barrel topology and domain B is found as a protuberant loop inserted into domain A^13,21^. While domains A and B comprise the enzyme active site, the C and E domains adopt β-sandwich folding and contain maltose-binding sites (MBS) for substrate binding^22^. Nevertheless, while the E domain belongs to the carbohydrate-binding module family 20 (E_CBM20_) and contains MBS1 and MBS2 involved in starch-binding, the C domain contains MBS3^23–25^. Domain D also adopts β-sandwich folding, but its function is to structurally connect the ABC architecture to the E_CBM20_ domain^26^. Both domains A and B include nine subsites (− 7 to + 2) that comprise the enzyme active site^27^. Thus, the starch substrate is arranged in a ring-shaped structure at the active site of CGTases and cleavage at subsites − 1 and + 1 by the conserved acidic catalytic triad Asp^x^, Glu^y^, and Asp^z^ from CSR II, III, and IV, respectively^13,18^. Simultaneously, residues at subsites + 2, − 2, and − 3 address the four catalytic activities of CGTases described above^28,29^, while residues at subsites − 4 to − 7 determine the CDs size specificity^13,30^; subsites − 6 and − 7 are absent in α-amylases^13^. Furthermore, a conserved aromatic central Tyr/Phe residue from CSR V (which is usually replaced by a nonaromatic residue in α-amylases) and a hydrophobic pair (Phe)/(Phe/Tyr) at subsite + 2, are essential for the cyclization activity of CGTases and to distinguish them from α-amylases^13,30,31^. Notably, structure-based protein engineering has shown that mutations in the active site change the specificity, allowing the conversion of CGTases to α-amylases^32–34^. CGTases from Gram-negative (G−) bacteria showed an unusual four-domain ABCE_CBM20_ distribution with the D domain absent^35^, and the classical E_CBM20_ domain is usually replaced in CGTases from archaea by a C-terminal E_arch_ domain with an unclear structure–function relationship^36^.

Carbohydrate metabolism via cyclodextrins (CM-CD) is an unusual microbial starch-converting pathway that involves synthesis, transportation, degradation, and metabolic assimilation of CDs^37,38^. Notably, although the CM-CD pathway is well described for the hyperthermophilic archaea *Thermococcus* sp., *Pyrococcus furiosus,* and *Archaeoglobus fulgidus*^39^, the descriptions from bacteria are limited to mesophilic G− *Klebsiella oxytoca*^37,40^ and G+ *Bacillus subtilis*^41,42^. Extracellular CGTases are the key enzymes that catalyze the first step of the CM-CD pathway by converting the surrounding starch substrate to CDs. In G− bacteria, CDs are subsequently internalized into the periplasm by a transmembrane cyclodextrin porin (CDP)^43^. The entry of CDs into the cytoplasm of bacteria and archaea occurs via a type I ATP-dependent ABC sugar importer system MdxEFG-(X/MsmX)^44^, which internalizes both cyclo/maltodextrin molecules^40,45,46^. Hence, sugar translocation into the cytoplasm is triggered by a dedicated MdxX ATPase in G− bacteria (CymD in *K. oxytoca*)^40^ or by a promiscuous MsmX ATPase in G+ bacteria and archaea^45,47,48^. The following reaction in the CM-CD pathway is the cleavage of CDs by a cytoplasmic cyclodextrinase (CDase, EC 3.2.1.54), resulting in maltose/maltooligosaccharides that are further degraded to glucose-1-phosphate (G1P) by an α-glucan phosphorylase (GP, EC 2.4.1.1)^37,38,42^. Finally, while glucose metabolism proceeds through the typical glycolytic pathway in *K. oxytoca* and *B. subtillis*^37,42^, a modified Embden-Meyerhof-Parnas (EMP) glycolytic pathway is found in archaea^38,39^.

Although CM-CD is considered a secondary pathway for starch breakdown and conversion in *K. oxytoca* and *B. subtilis*^37,42^, it is the main starch-converting pathway in sulfur-reducing hyperthermophilic archaea^38,39^. Similarly, extremely thermophilic bacteria [T_opt_ ≥ 70 °C^49^] such as Deinococcales, Thermotogales, and Thermoanaerobacterales that live in a wide variety of hot environments on Earth (*e.g.*, hydrothermal and geothermal vents) are capable of metabolizing a broad range of carbohydrates, including starch^50,51^. Nevertheless, because attention has been focused on the well-studied CGTases from Bacilli class bacteria, the identification and characterization of CGTases from extremely thermophilic bacteria have remained vague and are limited to *Thermoanaerobacter* spp.^52^, *Carboxydocella* sp.^53^, and *Thermoanaerobacterium thermosulfurigenes*^54^. Moreover, since the identification of CGTases for structure–function relationship studies has also been the central focus over the years, their functional role in a putative CM-CD pathway for extremely thermophilic bacteria remains obscure.

In this work, a novel group of CGTases from GH13_2 with an exceptional three-domain ABC architecture was detected by (meta)genome mining of microbial communities living in a wide variety of hot environments on Earth. Sequence analysis revealed that this group of CGTases belongs to the extremophilic Thermoanaerobacterales *Caldanaerobacter subterraneus* ssp., and *Thermoanaerobacter* spp. and shares ≤ 46% sequence identity with the CGTases characterized thus far. Sequence and comparative genomic analysis also showed that the three-domain ABC CGTase-encoding genes are exceptionally grouped in unrevealed gene clusters that encode the entire CM-CD pathway and several important proteins for prokaryotic cell functions. Together, functional studies of a representative member, CldA, combined with phylogenetic analysis revealed a new evolutionary path among CGTases and shed light on a nonclassical pathway for starch metabolism in Thermoanaerobacterales.

## Results

### Database mining for novel thermophilic CGTase enzymes

**Figure 1:**
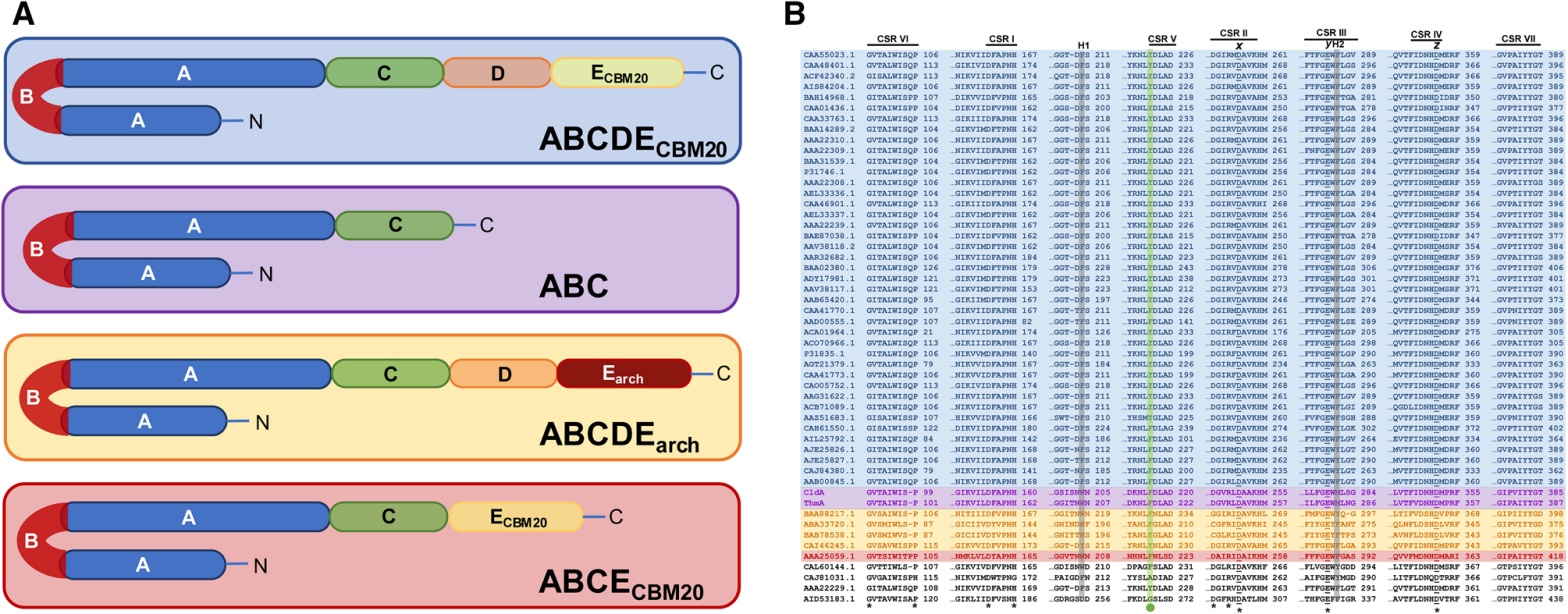
CGTases with different domain organizations. (**A**) Schematic representation of conventional five-domain ABCDE_CBM20_ CGTases (blue), five-domain ABCDE_arch_ CGTases (orange), and four-domain ABCE_CBM20_ CGTases (red), which are recognized by CAZy. Note that the novel group of 19 CGTases, (CldA/ThmA)-like enzymes from thermophilic *C. subterraneus* ssp. and *Thermoanaerobacter* spp., showed a three-domain ABC architecture (magenta). (**B**) Multiple amino acid sequence alignment of CGTases from GH13_2 with a conventional five-domain ABCDE_CBM20_ (blue), five-domain ABCDE_arch_ (orange), four-domain ABCE_CBM20_ (red), and three-domain ABC distribution (magenta), as well as maltogenic starch-acting enzymes (white). Note the CSR I-VII motifs showing functionally critical residues (asterisk) for the GH13 family. The underline indicates the conserved acidic catalytic triad Asp^x^, Glu^y^, and Asp^z^ from CSR II, III, and IV, respectively. The conserved aromatic central Tyr/Phe residue (green sphere) and the hydrophobic pair (Phe/Trp/Tyr)/(Phe/Tyr/Met) (H1 and H2 shadow boxes), which are essential for the cyclization activity of CGTases and to distinguish them from α-amylases are also showed^13,30,31^. The same color code is used in all other figures.

To identify putative CGTases involved in the CM-CD pathway of extremely thermophilic bacteria, a database mining approach was applied to ~ 130 public metagenomes of microbial communities from diverse thermophilic environments (Tables S1 and S2). Notably, a low number of putative CGTases were detected (14 hits in total; Table S1), which seems to be related to the rarity of the CM-CD pathway in extremely thermophilic bacteria living in starch-poor environments. Nevertheless, a CGTase-encoding gene (*cldA*) from Obsidian Pool hot spring metagenomic data at Yellowstone National Park was distinguished (Tables S1 and S2). Sequence analysis revealed that CldA consists of 524 residues and shares ≤ 42% sequence identity (100% query coverage) with the 51 characterized enzymes from GH13_2. A BLAST search in the nonredundant GenBank database revealed another three CldA-like sequences that share 98% average sequence identity with CldA (100% query coverage) and are annotated as hypothetical glycosidase/α-amylase enzymes in eight available genomes from several Thermoanaerobacterales subspecies of G+ thermophilic *Caldanaerobacter subterraneus* (Table S3). Although *C. subterraneus* subspecies (T_opt_ of 60–85 °C) are found in various extremophilic environments^55–57^, they natively live in the Obsidian Pool hot spring at Yellowstone National Park^58^. Sequence analysis also revealed that CldA exhibits a 21-residue N-terminal signal peptide, _1_M**RKNFK**AFVALFAAILLFFSG**C**, which contains a positively charged tail, _2_RKNFK, followed by a hydrophobic core region that ends with the conserved Cys22 (boldface residues) typical for the cleavage site of signal peptidase type II (SPII)^59^. In agreement with this observation, the extracellular glycoside hydrolases of the GH13 family from G+ bacteria are translocated from the cytoplasmic membrane through the general secretion (Sec) system^60,61^. Because CldA and CldA-like enzymes displayed an unusual short-form sequence compared to conventional five-domain CGTases (Figs. 1A and S1), a functional domain analysis was conducted. Remarkably, CldA showed an atypical three-domain ABC distribution compared to CGTases with either conventional five-domain ABCDE_CBM20_, five-domain ABCDE_arch_, or four-domain ABCE_CBM20_ distribution (Fig. 1A). Thus, the mature form of CldA consists of catalytic AB domains (residues 22–434) and the starch-binding C domain (residues 434–524) at the C-terminal region (Figs. 1A and S1). Sequence alignment of CldA with the 51 characterized CGTases from GH13_2 revealed the presence of CSR I-VII motifs from the GH13 family (Fig. 1B), including the highly conserved catalytic triad Asp250, Glu279, and Asp351 from CSR II, III, and IV, respectively (Fig. 1B), which is involved in glycoside bond cleavage^18^. Furthermore, both the conserved aromatic central Phe216 residue from CSR V (which is usually replaced by a nonaromatic residue in α-amylases) and the pair of hydrophobic residues Trp204/Met281, which are critical in sugar chain circularization for CD formation, were observed (Fig. 1B)^31,62^. Interestingly, while Met281 belongs to CSR III, Trp204 is found in a _199_GSISN**W**N motif. Although CldA was found in G+ bacteria, both _199_GSISN**W**N and CSR VI motifs were observed in CGTases from archaea and G− (Fig. 1B). Hence, the presence of these unique three-domain ABC CGTases in the *Caldanaerobacter* genus (Table S3) also suggests a putative CM-CD pathway for starch metabolism.

### Functional characterization of CldA

**Figure 2:**
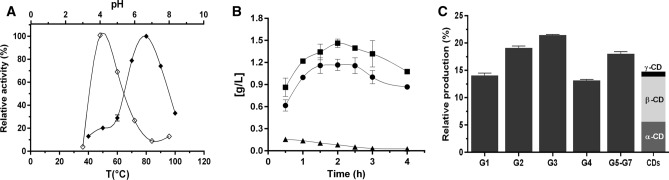
CldA enzymatic assay. (**A**) Effect of temperature (filled diamonds) and pH (empty diamonds) on CGTase activity. (**B**) Production of α-CD (circles), β-CD (squares) and γ-CDs (triangles) from 50 g L^−1^ soluble starch by the action of CldA at 75 °C and pH 4.0 for 4 h. (**C**) The relative production of end products from 50 g L^−1^ soluble starch after 2 h of reaction at 75 °C and pH 4.0. Note that G5-G7 is the sum of the linear oligosaccharides maltopentaose, maltohexaose, and maltoheptaose. The error bars indicate the standard deviation of three replicates.

The recombinant CldA enzyme was successfully produced in *Escherichia coli* to evaluate CGTase activity. The mature form of CldA consists of 511 residues with a calculated molecular mass of 58.4 kDa, including a C-terminal His_6_-tag sequence without the N-terminal signal peptide. Protein purification was performed by a heat treatment procedure and nickel-affinity chromatography followed by size-exclusion chromatography (SEC)-dynamic light scattering (DLS) coupled experiments (Fig. S2A), resulting in a purification yield of ~ 45 mg CldA from 1 L of culture. Purified recombinant CldA showed a molecular mass of 58.5 kDa in the SEC-DLS analysis with an optimal monodispersity (Mw/Mn = 1.02), showing that the biological assembly is monomeric (Fig. S2A). CldA also showed a molecular mass of ~ 58 kDa on SDS-PAGE (Fig. S2B) and a theoretical isoelectric point (pI) of 5.7. CldA displayed cyclization activity over a broad range of temperatures from 40 to 100 °C and pH ranges from 4 to 8 (Fig. 2A), using soluble starch as the substrate. Furthermore, CldA reached more than 65% relative cyclization activity at acidic pH (4–5) and high temperatures (70–90 °C) (Fig. 2A). CldA also displayed a half-life (*t*_1/2_) of 25.5 min at 80 °C and extraordinary thermostability at 70 °C (*t*_1/2_ = 63.4 h) (Fig. S3). CD production was monitored over time by incubating CldA with 50 g L^−1^ soluble starch at 75 °C and pH 4. The production of α-, β-, and γ-CDs increased over time, achieving the maximum yield of total CDs (2.72 ± 0.06 g L^−1^) after 2 h of incubation (Figs. 2B and S5). The proportion of α- and β-CDs (34:62) was relatively conserved over time with minor γ-CD production (Fig. 2B,C), revealing that the CldA enzyme is a β-CGTase. Nevertheless, while CldA displayed a specific β-cyclization activity of 51.26 ± 6.3 U mg^−1^, it exhibited an unusual high hydrolytic activity of 405.40 ± 5.4 U mg^−1^. According to the latter, CldA yielded as the primary products those related to the hydrolysis of soluble starch, such as linear oligosaccharides with different degrees of polymerization (G3–G7) and the fermentable sugars maltose (G2) and glucose (G1) (Figs. 2C and S4). All products synthesized by the action of CldA from soluble starch were confirmed by HPLC and mass spectrometry analysis (Figs. S4 and S5).

### Discovery of a novel group of three-domain ABC CGTases

**Figure 3:**
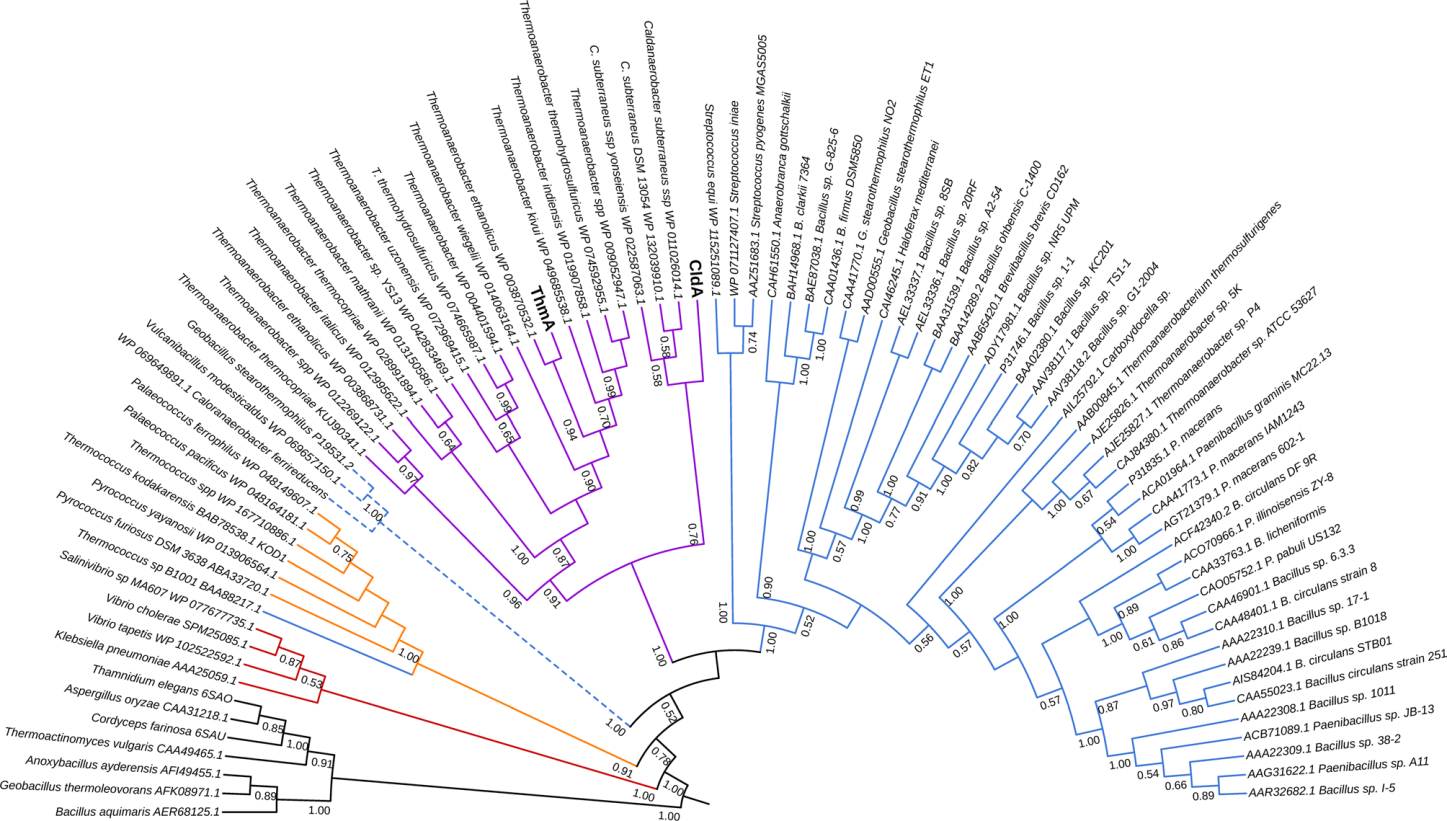
Phylogenetic analysis of novel three-domain ABC CGTases. Evolutionary relationships were determined by the maximum likelihood method based on the WAG + G model using the full amino acid sequences of 78 CGTases, including the 48 characterized CGTases from GH13_2 recognized in the CAZy database, 19 three-domain ABC (CldA/ThmA)-like CGTases, and 11 putative CGTases. The sequences of 7 α-amylases from GH13 were used as an outgroup. The conventional five-domain ABCDE_CBM20_ CGTases (blue), five-domain ABCDE_arch_ CGTases (orange), four-domain ABCE_CBM20_ CGTases (red), and the novel group of 19 three-domain ABC CGTases, (CldA/ThmA)-like enzymes from thermophilic *C. subterraneus* ssp. and *Thermoanaerobacter* spp. (magenta) were observed in four different clades. The ABCDE_CBM20_ maltogenic starch-acting enzymes (blue dashed line) and α-amylases (black branch) from GH13_2 and GH13, respectively, are also shown in two different clades. Note that while the α-amylases from *Aspergillus oryzae* and *Cordyceps farinosa* belong to the GH13_1 subfamily, the α-amylases from bacteria showed an unassigned GH13 subfamily. Bootstrap values (1000 iterations) are indicated for each node. Only bootstrap values above 50% were shown. The tree was drawn using iTOL v4 (http://itol.embl.de).

To identify additional three-domain ABC CGTases, a database mining approach was also applied to ~ 30 public metagenomes of microbial communities from the Obsidian Pool hot spring (Table S2), using the CldA sequence as a template. The database mining approach revealed a homologous CGTase-encoding gene (*thmA*) that codifies for a 526-residue CGTase (Table S3) sharing 80% sequence identity with CldA (100% query coverage) (Fig. S1). Functional domain analysis showed that ThmA is a three-domain ABC CGTase exhibiting the highly conserved Asp252/Glu281/Asp353 catalytic triad, the conserved aromatic central Phe218, and the pair of hydrophobic residues Trp206/Met283 (Fig. 1B). A BLAST search in the GenBank database of the ThmA enzyme showed 100% sequence identity with a putative glycosidase from *Thermoanaerobacter ethanolicus*. Furthermore, 14 putative ThmA-like sequences encoded in 16 genomes from several Thermoanaerobacterales subspecies of G+ thermophilic *Thermoanaerobacter* spp. were also found (Table S3). A subsequent BLAST search in the GenBank database confirmed that the 19 three-domain ABC (CldA/ThmA)-like CGTases (Table S3) belong to *C. subterraneus* ssp. and *Thermoanaerobacter* spp., respectively. Furthermore, CldA and ThmA share only 38% average sequence identity with three characterized five-domain ABCDE_CBM20_ CGTases (100% query coverage for ABC domains) from *Thermoanaerobacter* spp.^52^, confirming that both three-domain CldA/ThmA CGTases are not truncated forms from conventional five-domain CGTases. Accordingly, to determine the evolutionary relationship among this novel group of three-domain CGTases with all characterized CGTases from GH13_2, a phylogenetic analysis was conducted, including seven α-amylases from GH13 as an outgroup. The analysis showed that the CGTases were distributed in five phylogenetic groups that presented a bootstrap value of 100% (Fig. 3). The four-domain ABCE_CBM20_ CGTases from G−, five-domain ABCDE_arch_ CGTases from archaea, and conventional five-domain ABCDE_CBM20_ CGTases from the well-studied G+ Bacilli class bacteria were observed in three different clades. Nevertheless, it has been shown that the five-domain ABCDE_CBM20_ configuration is not unique to CGTases from G+, as has been observed in the thermophilic CGTase from archaea *Thermococcus* sp. B1001 and the halophilic CGTase from archaea *Haloferax mediterranei*. A fourth clade comprises maltogenic starch-acting enzymes from GH13_2, which showed sequence and structural homology with CGTases was previously described elsewhere^63,64^. Notably, the 19 three-domain ABC (CldA/ThmA)-like CGTases were clustered together in a fifth new monophyletic group that is well supported by a bootstrap value of 100%, revealing a novel group of CGTases that is separated from the four conventional GH13_2 clades (Fig. 3). Identical phylogenetic results were obtained using the full amino acid sequence (Fig. 3) or solely the amino acid sequence of the minimal functional core ABC (Fig. S6) for all sequences analyzed.

### Database mining for the CM-CD pathway in Thermoanaerobacterales

**Figure 4:**
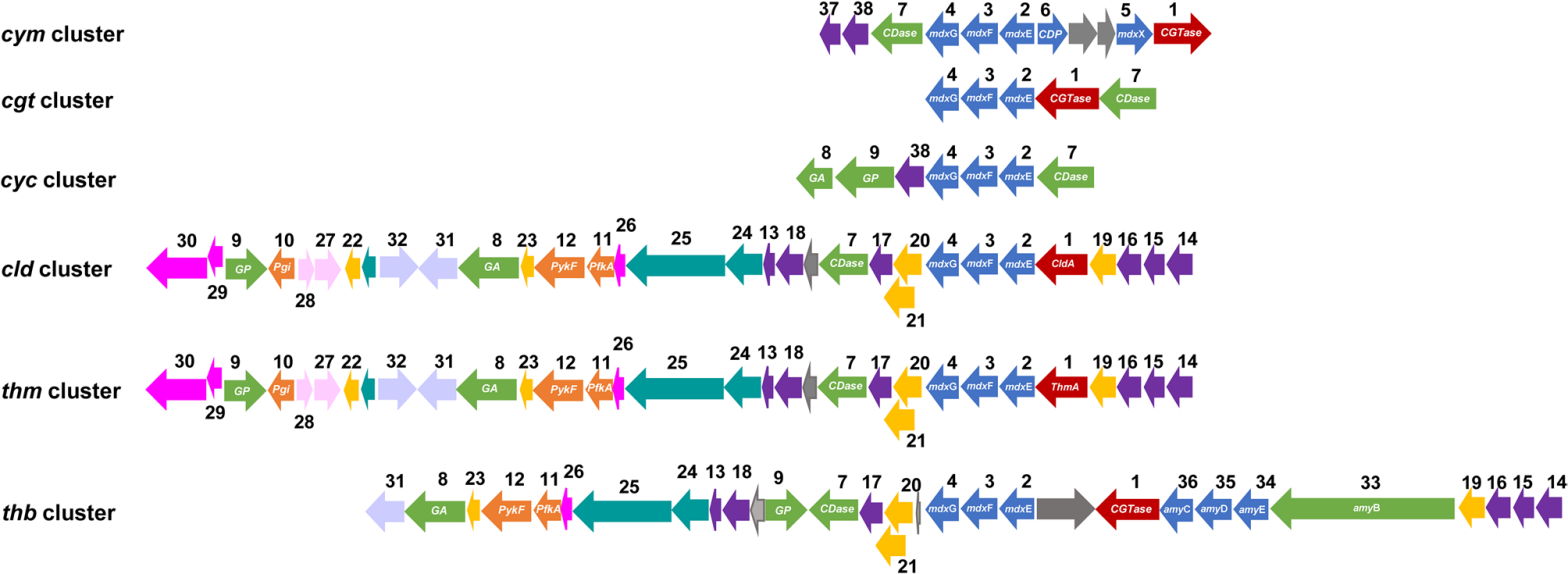
Comparative view of the gene clusters involved in the CM-CD pathway. Note the genetic organization of the CM-CD gene clusters from *K. oxytoca* (*cym*), *Thermococcus* sp. (*cgt*), *B. subtilis* (*cyc*), *C. subterraneus* ssp. (*cld*), *Thermoanaerobacter* spp. (*thm*), and *Thermoanaerobacterium* spp. (*thb*). Additionally, note the protein-encoding genes involved in the four steps of the CM-CD pathway. (i) Synthesis: CGTases (1, red). (ii) Translocation/Internalization: MdxE (2), MdxF (3), and MdxG (4) in blue. While the MdxX (5) and CDP (6) from G− *K. oxytoca* (*cym*) are also blue, the putative *msm*X-encoding gene is not included. (iii) Degradation: CDase (7), GA (8), and GP (9) in green. (iv) Metabolic assimilation: Pgi (10), PfkA (11), and PykF (12) in orange. AmyB (33) and the AmyEDC transporter system (34–36) from *Thermoanaerobacterium* spp. (*thb*), and the putative transcriptional regulator of the ABC transporter system from *cym*/*cyc* (37–38) are shown. Note the five groups of protein-encoding genes that are essential for several prokaryotic cell functions: (i) HPr (13), PolIIIα (25), and the CBS domain/Bateman module (24) for carbon catabolite regulation, bacterial genome replication, and sensing cellular energy status, metal ion concentration, and ionic strength. (ii) MurB (14), PHP (15), RapZ (16), RodZ (17), and WhiA (18) for cell wall biogenesis, sporulation, and cell division. (iii) feruloyl esterase (22), 2-phospho-l-lactate transferase (19), the enzyme system (R)-2-hydroxyglutaryl-CoA dehydratase (20, 21), and 4-hydroxy benzoyl-CoA thioesterase (23) for oxidative stress defense, degradation of aromatic compounds, and fatty acid metabolism. (iv) signal-transducing protein PII (26), methylenetetrahydrofolate reductase (29), methionine synthase (30), PepT (27) and the anaerobic transcriptional activator *fnr* (28) for amino acid metabolism. (v) tRNA(m^5^U_54_)methyltransferase (31) and MATE (32) for tRNA maturation and detoxification. Genes of unknown function are in gray. Abbreviations are listed in Table S4.

Because *C. subterraneus* is the only species formally recognized from the *Caldanaerobacter* genus^55,65^, the eight publicly assembled and draft genomes from the four subspecies of *C. subterraneus* ssp. were examined (Table S3), focusing on the gene clusters where the (CldA/ThmA)-like-encoding genes are located. Strikingly, a gene cluster of 30 genes (*cld*) of the 1130 total gene clusters encompassing the core genome from the *Caldanaerobacter* genus^66^ was identified in the complete assembled scaffolds from *C. subterraneus* ssp. (Fig. 4, Table S4). Sequence analysis of the *cld* gene cluster predicts several proteins of the CM-CD pathway: a putative type I ATP-dependent ABC transporter system, MdxEFG (CldEFG), with the *cld*EFG gene cassette located immediately downstream of the *cld*A-like-encoding gene, as well as the three cytoplasmic enzymes CDase, GP, and a glucoamylase from GH15 (GA, EC 3.2.1.3). Predictive functional analysis showed that the *cld*E-encoding gene from the *cld*EFG gene cassette codifies for a periplasmic MdxE cyclo/maltodextrin-binding protein that shares 40% average sequence identity (100% query coverage) with the MdxE proteins from G+ *Thermoactinomyces vulgaris* (*Tvu*CMBP, PDB ID: 2DFZ^47^) and G+ *Alicyclobacillus acidocaldarius* (MalE)^46^. Sequence analysis also revealed that CldE exhibits a 24-residue N-terminal signal peptide, _1_MKKYSKILALLTAMVFVLSIALTG**C**G, containing the conserved Cys25 (boldface residue), which is essential to anchor the MdxE proteins from G+ and archaea to the cytoplasmic membrane outer surface via an N-terminal lipid moiety that is covalently bound to the Cys residue^67^. The *cld*FG-encoding genes from the *cld*EFG gene cassette (Fig. 4, Table S4) encode two putative ABC transporter permease subunits, CldF and CldG, that share 40% average sequence identity (100% query coverage) with the CymFG/CgtDE/YvfL-YvfM/MalFG permease subunits from the MdxEFG transporter system of *K. oxytoca*^40^, *Thermococcus* sp.^45^, *B. subtilis*^41^, and *A*. *acidocaldarius*^46^, respectively.

The putative CDase encoded in the *cld* gene cluster shares 88% sequence identity with the functionally characterized CDase from *Thermoanaerobacter thermohydrosulfuricus* (NCBI ID: AAA23219.1), which hydrolyzes CDs to yield maltodextrins G2 and G1^68^. Thus, while CDase linearizes CDs into the cytoplasm, the resulting dextrins are converted mainly into G1/G1P by the GA/GP enzymes encoded in the *cld* gene cluster (Fig. 4, Table S4). Both GA and GP enzymes have been functionally characterized elsewhere^69,70^. Furthermore, several proteins of the EMP pathway from *C. subterraneus* ssp., such as phosphoglucose isomerase (Pgi, EC 5.3.1.9), 6-phosphofructokinase (PfkA, EC 2.7.1.11), and the functionally characterized pyruvate kinase (PykF; EC 2.7.1.40)^71^, were also found in the *cld* gene cluster (Fig. 4, Table S4). Similarly, the genomes of all Thermoanaerobacterales were also tested using an expanded searching cross-family algorithm to identify additional CM-CD-encoding gene clusters. Remarkably, two gene clusters (*thm* and *thb*) involved in the CM-CD pathway were also identified in the assembled genomes from *Thermoanaerobacter* spp. and *Thermoanaerobacterium* spp., respectively (Fig. 4, Table S4). Sequence analysis of the *thm* and *thb* gene clusters predicts several proteins of the CM-CD pathway: a putative type I ATP-dependent ABC transporter system, MdxEFG (CldEFG), and the three cytoplasmic enzymes CDase, GP, and GA. Nevertheless, while the *thm* gene cluster contains three-domain ABC ThmA-like CGTases, the *thb* gene cluster contains a conventional five-domain ABCDE_CBM20_ CGTase. In addition, although the Pgi-encoding gene was absent in the *thb* gene cluster, the critical enzymes for the EMP pathway were encoded in both the *thm* and *thb* gene clusters (Fig. 4, Table S4).

Remarkably, sequence analysis of the *cld*/*thm*/*thb* gene clusters also revealed the presence of 18 protein-encoding genes that are essential for prokaryotic cell functions (Fig. 4, Table S4), such as the functionally and structurally characterized phosphotransferase HPr (PDB ID: 3LE5), which is a key enzyme for carbon catabolite regulation in *C*. *subterraneus* ssp. *tengcongensis*^72^, *Thermoanaerobacter* spp.^73^, and *Thermoanaerobacterium* spp.^74^, as well as a DNA polymerase III (PolIIIα, EC 2.7.7.7) responsible for bacterial genome replication^75^, which is preceded by a putative CBS domain/Bateman module involved in sensing cellular energy status, metal ion concentration, and ionic strength^76,77^. The second group of putative proteins of the *cld*/*thm*/*thb* gene clusters is involved in cell wall biogenesis, sporulation, and cell division: (i) UDP-N-acetylmuramate dehydrogenase (MurB, EC 1.3.1.98) is involved in the biosynthesis of bacterial cell wall peptidoglycan^78^, (ii) histidinol phosphatase (PHP) is required in the phosphorelay system to regulate the biosynthesis of cell wall-associated polysaccharides^79^, (iii) RapZ regulator is implicated in the RNA-mediated regulatory network of glucosamine biogenesis^80^, (iv) the transmembrane RodZ protein is a key protein in cell elongation (elongasome) and cell division^81,82^, and (v) the sporulation transcription WhiA regulates cell differentiation^83,84^. The third group of proteins is essential for oxidative stress defense, degradation of aromatic compounds, and fatty acid metabolism: (i) the functionally characterized feruloyl esterase (EC 3.1.1.73) from *C. subterraneus* ssp. *tengcongensis*, which can hydrolyze esterified phenolic acids from xylan and pectin^85^, (ii) 2-phospho-l-lactate transferase (EC 2.7.8.28) involved in the biosynthesis of redox coenzyme F_420_, which is important for the redox transformations of cell wall lipids, degradation of aromatic/xenobiotic compounds, and neutralization of oxidative and nitrosative stress^86,87^, (iii) the two components E1 (activator) and E2 (dehydratase) of the enzyme system (R)-2-hydroxyglutaryl-CoA dehydratase (EC 4.2.1.167), which is involved in glutamate metabolism via butyrate fermentation in G+ bacteria^88^, and (iv) putative 4-hydroxy benzoyl-CoA thioesterase, which can hydrolyze fatty acyl-CoA thioesters^89^. The fourth group of putative proteins is implicated in amino acid metabolism: (i) signal-transducing protein PII involved in the regulation of nitrogen metabolism via glutamine/glutamate cycle^90^, (ii) methylenetetrahydrofolate reductase (EC 2.1.1.13), and methionine synthase (EC 1.5.1.20), which are both involved in methionine biosynthesis via methyltetrahydrofolate (methyl-THF), and (iii) tripeptide aminopeptidase T (PepT; EC 3.4.11.4), which is preceded by its anaerobic transcriptional activator *fnr*^91^ and is only included in the *cld*/*thm* gene clusters. Finally, the putative tRNA(m^5^U_54_)methyltransferase (EC 2.1.1.190) and a multiantimicrobial extrusion protein (MATE), which might be involved in tRNA maturation and detoxification, respectively^92–94^, are also encoded in the *cld*/*thm*/*thb* gene clusters. Although G− *K. oxytoca*, archaea *Thermococcus* sp., and G+ *B. subtilis* arranged the proteins involved in the CM-CD pathway in three similar gene clusters, *cym*, *cgt*, and *cyc*, respectively (Fig. 4), none of the latter protein-encoding genes for prokaryotic cell functions and the proteins for the EMP pathway are encoded near their CM-CD gene clusters. The proteins encoded in the *cld*/*thm* gene clusters (Fig. 4) are shown in Table S4.

## Discussion

Traditionally, the five-domain ABCDE_CBM20_ organization has been considered the central architecture of CGTases, with the only few exceptions for five-domain ABCDE_arch_ CGTases from archaea and four-domain ABCE_CBM20_ CGTases from G−, highlighting the recurrence of both the ABC core structure and the E_CBM20_/E_arch_ domain in the overall CGTase fold. Here, a database mining approach allowed the identification of a novel group of three-domain ABC (CldA/ThmA)-like CGTases from G+ thermophilic *C. subterraneus* ssp. and *Thermoanaerobacter* spp., respectively, which exhibit a unique CGTase domain distribution that is different from that seen in all other CGTases characterized thus far (Fig. 1A). Notably, although the (CldA/ThmA)-like enzymes displayed a distinctive active site for CGTases with the presence of all CSR I-VII motifs from the GH13 family (Fig. 1B), the three-domain ABC architecture is not commonly associated with conventional CGTases. The functional characterization of a representative member, the three-domain ABC CldA, revealed that regardless of whether β-CD is synthesized as the major cyclization product from the starch substrate under the assay conditions, cyclization does not appear to be the main activity of the enzyme (Fig. 2). Accordingly, the production of fermentable sugars, dextrins, and functional CDs from the starch substrate by the action of extracellular (CldA/ThmA)-like CGTases seems to be a reasonable adaptation to diversify products and increase the probability of survival in extremely hot environments with low starch and nutrient concentrations. Compared with the CldA enzyme, similarly increased hydrolytic and decreased cyclization products have been observed for several CGTases from archaea and thermophilic bacteria^36,54^.

The identification of this novel group of enzymes showed for the first time that the three-domain ABC organization represents the minimal functional core structure for CGTases and confirmed previous studies suggesting that the C-terminal region of CGTases has been acquired through evolutionary processes^15,35,95^. Indeed, while the raw starch-binding E_CBM20_ domain is observed in several GH families^24,96,97^, both the E_arch_ domain with an unknown structure–function relationship and the connecting D domain are unique to CGTases^15,35,95^. Interestingly, the three-domain CGTases clustered together in a new monophyletic group that diverged as a novel evolution path among conventional CGTases. Hence, while the four-domain CGTases from G− separated early from the rest of CGTases, the three-domain CGTases and both groups of five-domain CGTases diverged later from a common ancestor. This observation also indicates that three-domain CGTases are not truncated forms from either of the two groups of five-domain CGTases, and the minimal ABC framework of the (CldA/ThmA)-like enzymes from Thermoanaerobacterales is not the common ancestor of all CGTases (Fig. 3).

**Figure 5:**
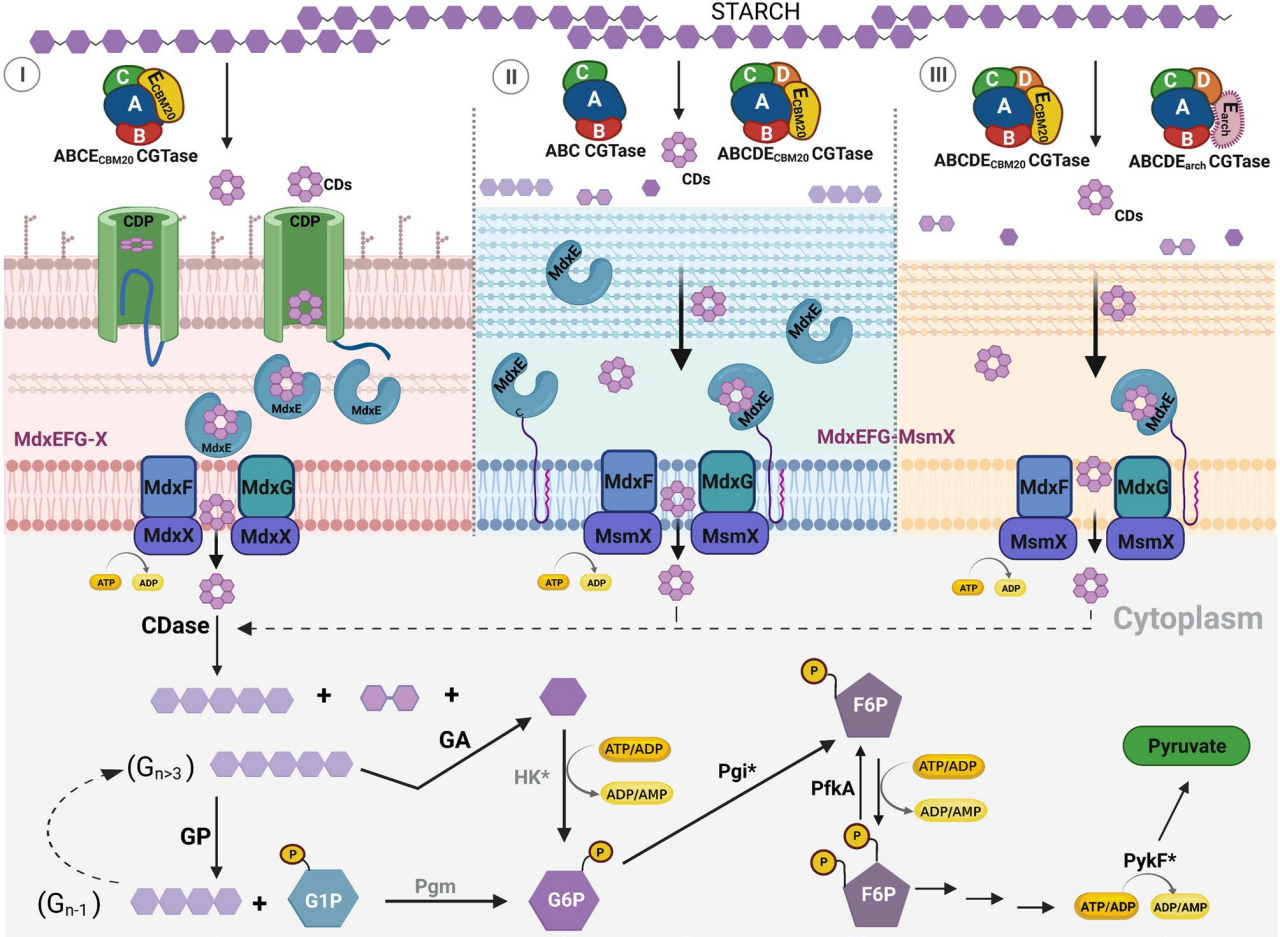
Proposed CM-CD pathway in G− bacteria (I), G+ bacteria (II), and archaea (III). Note the proteins involved in the four steps of the CM-CD pathway. (i) Synthesis: four-domain CGTases in G−, three- and conventional five-domain CGTases in G+ , and five-domain CGTases in archaea with either E_CBM20_/E_arch_ domains at the C-terminal region. (ii) Translocation/Internalization: MdxEFG-(X/MsmX) transporter system. The CDP in G− is also shown. Note that while the cyclo/maltodextrin-binding protein MdxE is an untethered component of the periplasmic space in G−, it is predicted to be anchored to the cytoplasmic membrane outer surface via a lipid moiety in G+ and archaea. Although the MdxX enzyme is a dedicated ATPase in G−, MsmX is a promiscuous ATPase in G+ and archaea. Cyclo/maltodextrin translocation into the cytoplasm by the two permease subunits MdxFG is triggered by the ATPase activity of MdxX/MsmX. (iii) Degradation: CDase, GA, and GP. Hexagons represent individual glucose molecules. (iv) Metabolic assimilation: Pgi, PfkA, and PykF. While Pgm and HK are not included in the CM-CD gene clusters of Fig. 4, the asterisks in HK*, Pgi*, and PykF* represent the modified EMP pathway in archaea. This figure was created with http://BioRender.com.

In addition to the phylogenetic analysis, the presence of this novel group of three-domain CGTases suggests a role in starch metabolism. Nevertheless, Thermoanaerobacterales are obligate anaerobic Clostridia class bacteria with low genomic G + C content capable of thriving in various hot environments on Earth, such as geothermal fields, submarine hydrothermal vents, and oil reservoirs^57,98^, which are expected to be starch-poor environments. Consequently, genomic gene clustering analysis against 246 Thermoanaerobacterales genomes allowed the identification of only three gene clusters involved in the CM-CD pathway, *cld*, *thm*, and *thb*, from the Thermoanaerobacteracea family (*C. subterraneus* ssp. and *Thermoanaerobacter* spp.) and from Thermoanaerobacterales family III (*Thermoanaerobacterium* spp.), respectively, confirming the rarity of the pathway. Thus, while the three-domain (CldA/ThmA)-like-encoding genes belong to the *cld* and *thm* gene clusters, respectively, the *thb* gene cluster contains a conventional five-domain CGTase-encoding gene (Fig. 4). Based on comparisons with G− *K. oxytoca*, archaea *Thermococcus* sp., and G+ *B. subtilis,* which arranged the proteins involved in the CM-CD pathway in three similar gene clusters, *cym*, *cgt*, and *cyc*, respectively (Fig. 4), the first step of the CM-CD pathway in Thermoanaerobaterales involves converting the surrounding starch substrate to CDs catalyzed by secreted three- and five-domain CGTases (Fig. 5). As previously established by X-ray crystallography studies, the resulting CDs are then internalized into the periplasm by a transmembrane β-barrel CDP in G− *K. oxytoca* (CymA, PDB ID: 4V3G), which mediates the passive diffusion of CDs through the perturbation of electrostatic interactions of the N-terminal region with the β-barrel wall of CDP. Therefore, the 15 N-terminal residues of CymA are expelled from the barrel through a ligand-expelled gate mechanism, allowing the diffusion of CDs into the periplasmic space^43^. As expected, owing to the differences in the cell wall composition between G+ and G− bacteria, the outer-membrane translocation of CDs in G+ remains uncertain, as no putative CDP was detected in the extensive data mining analysis using the CymA sequence. However, sequence analysis revealed that the putative MdxEFG transporter system, CldEFG, which is present in all three *cld*/*thm*/*thb* gene clusters (Fig. 4), appears to be translocating cyclo/maltodextrin molecules through the peptidoglycan layer and subsequently internalizing them into the cytoplasm (Fig. 5). Similar MdxEFG transporter systems, which translocate cyclo/maltodextrin molecules into the cytoplasm, have been described in G− *K. oxytoca* (CymEFGD)^40^, archaea *Thermococcus* sp. (CgtCDE)^45^, G+ *B. subtilis* (CycB-YvfL-YvfM)^41^, and *A. acidocaldarius* (MalEFG)^46^ (Fig. 5). Accordingly, translocation through the MdxEFG transporter system initiates when MdxE binds the cyclo/maltodextrin molecules synthesized by CGTases. The crystal structure of the cyclo/maltodextrin-binding protein MdxE, *Tvu*CMBP, showed the classical architecture of bacterial sugar-binding proteins, consisting of two domains that are joined by a hinge region, which surrounds a sugar-binding site located at a cleft formed by the two domains^47^. Hence, *Tvu*CMBP binds cyclo/maltodextrin molecules and undergoes substantial conformational changes to transit from the open to the sugar transporter closed conformation to release them into a transmembrane protein complex composed of the two putative permease subunits MdxF and MdxG. Notably, it has also been shown that MdxE, MalE, from G+ *A. acidocaldarius* is anchored to the cytoplasmic membrane outer surface via a lipid moiety that is covalently bound to an N-terminal cysteine residue, so it can be distributed throughout the cell wall to scavenge the surrounding cyclo/maltodextrin molecules that are synthesized by CGTases to release them into the MdxFG system^46^. In contrast, the cyclo/maltodextrin-binding protein CymE from G− *K. oxytoca* is an untethered component of the periplasmic space that binds the cyclo/maltodextrin molecules diffused through the transmembrane CDP to release them into the MdxFG system^40^ (Fig. 5). Owing to modifications in the cell wall composition and the absence of a transmembrane CDP in G+ bacteria, differences between MdxE proteins from G+ and G− bacteria are typical features that distinguish sugar-binding proteins from type I ATP-dependent ABC transporter systems^44^. Thus, because CldE also includes the N-terminal Cys25 residue that covalently binds to a lipid moiety for anchoring to the cytoplasmic membrane outer surface, translocation through the CldEFG transporter system encoded in the *cld*/*thm*/*thb* gene clusters appears similar to the translocation mechanism of the MdxEFG transporter system, MalEFG, from G+ *A. acidocaldarius* (Fig. 5). In the next step, cyclo/maltodextrin translocation into the cytoplasm occurs through a conformational change of the two permease subunits MdxFG triggered by the ATPase activity of MdxX/MsmX. Accordingly, the MdxEFG-X transporter system from G− *K*. *oxytoca* includes a dedicated intracellular pair of ATP-binding components encoded in the same *cym* gene cluster by *mdx*X (CymD) (Fig. 4), which is coupled to the two permease subunits CymFG^37,40^ (Fig. 5). In contrast, the CgtCDE, CycB-YvfL-YvfM, and MalEFG transporter systems include a promiscuous MsmX ATPase with the same function as MdxX but exhibiting different nonspecific hydrophobic interactions with several transmembrane complexes, promiscuously energizing multiple sugar importers^48,99^ (Fig. 5). The latter observation is quite common in various carbohydrate ABC transporter systems from G+ bacteria^99^. Notably, additional data mining analysis revealed that *C. subterraneus* ssp., *Thermoanaerobacter* spp., and *Thermoanaerobacterium* spp. encoded a putative MsmX ATPase (NCBI ID: WP_011026113.1, WP_003866589.1, and WP_015311043.1, respectively) (Table S4), which completes the putative type I ATP-dependent ABC transporter system, CldEFG-MsmX, from the Thermoanaerobacterales order (Fig. 5). As expected, the *msm*X-encoding gene is distally located from the *cld*/*thm*/*thb* gene clusters and shares 64% sequence identity with the functionally and structurally characterized MsmX from *B. subtilis* (NCBI ID: WP_003242648.1, PDB ID: 6YIR)^100^. The following step of the CM-CD pathway involves several enzymes encoded in the *cld*/*thm*/*thb* gene clusters (Fig. 4, Table S4), which are essential for the cleavage and degradation of CDs into the cytoplasm through the EMP pathway (Fig. 5). Thus, while the linearization of CDs by CDase produces G1 and G2 molecules for the EMP pathway, dextrins (G_n>3_) are either converted into G1 or G1P (with the release of G_n-1_ dextrin) by the action of GA and GP enzymes, respectively. Both G1 and G1P molecules could be converted into G6P by the action of ADP-dependent hexokinase (HK) and phosphoglucomutase (Pgm), respectively, to also be metabolized through the EMP pathway (Fig. 5). Furthermore, since the putative Pgi and PfkA enzymes and the functionally characterized PykF^71^ of the EMP pathway are encoded exceptionally near the protein-encoding genes for (CldA/ThmA)-like CGTases, the CldEFG transporter system, CDase, GP, and GA enzymes (Fig. 4, Table S4), the entire CM-CD pathway from the Thermoanaerobacterales order is revealed (Fig. 5). Thus, while the synthesis of CDs might have a physiological role as functional amphipathic toroids^6,8,10^, the resulting G2 and G1 molecules, as well as the G3-G7 dextrins, could serve as a simple carbon source (Fig. 5).

Interestingly, the entire CM-CD pathway is encoded along with several essential proteins for G+ cell functions, such as DNA replication, carbon catabolite regulation, tRNA maturation, biogenesis, sporulation, and cell division (Fig. 4, Table S4), suggesting that extracellular heat-resistant CGTases could play a leading role in the metabolism of Thermoanaerobacterales. Moreover, the presence of protein-encoding genes related to extreme thermophilic metabolism, such as oxidative stress defense, degradation of aromatic compounds, fermentation, and fatty acid and amino acid metabolism (Fig. 4, Table S4), also indicates that the physiological role of heat-resistant CGTases in product diversification seems to be a convenient adaptation to survive in hot starch-poor environments. Accordingly, the relevance of CGTases during starch metabolism can be supported by early observations of *Thermoanaerobacterium* spp.^101^, in which the secreted thermophilic α-amylase/amylopullulanase AmyB was found to hydrolyze a variety of α-(1,4)- and α-(1,6)-glucans^102^, acting together with an ABC maltose/maltotriose importer (*amy*EDC)^101^. Notably, the *amy*BEDC gene cluster is located immediately upstream of the conventional five-domain CGTase (formerly named AmyA)^54^ (Fig. 5) encoded in the *thb* gene cluster (Fig. 4). Thus, AmyB and the five-domain CGTase seem to play a cooperative role, as it has been shown that the transcription of the *amy*BEDC gene cluster and the CGTase-encoding gene is induced by maltose or starch as carbon sources^101^. Likewise, the deduced promoter sequences of *cld*A/*thmA* genes, 5′-TGCACT-17 bp-TAATAT, and 5′-TTTCGA-17 bp-CATATT, showed similarity to the σ-dependent consensus promoters of the *amy*ABEDC gene cluster^101^. However, the database mining analysis revealed that the AmyB-like enzyme from *C. subterraneus* ssp. and *Thermoanerobacter* spp. is not encoded near the *cld/thm* gene clusters (Fig. 4), indicating that the secreted three-domain CGTases are the main starch-acting enzymes of both gene clusters, highlighting their importance for product diversification on these microorganisms.

In summary, this is the first identification of a novel group of CGTases with an uncommon three-domain ABC organization, which further established a new evolutionary path among CGTases. These novel enzymes were detected in two gene clusters, *cld* and *thm*, from extremely thermophilic Thermoanaerobacterales *C. subterraneus* ssp. and *Thermoanaerobacter* spp., as part of a CM-CD pathway involved in the synthesis, transportation, degradation, and metabolic assimilation of CDs from starch. These findings were extended to Thermoanaerobacterales *Thermoanaerobacterium* spp., which also showed a CM-CD pathway not previously described but governed by a conventional five-domain CGTase encoded in the *thb* gene cluster. In contrast to the secondary role of the CM-CD pathway in mesophilic bacteria, the remarkable product diversification catalyzed by the three-domain CGTases suggests that they could play a critical role in the carbohydrate metabolism of *C. subterraneus* ssp. and *Thermoanaerobacter* spp. Future X-ray crystal structure determination, structure-based protein engineering, and kinetic studies of CldA will offer an opportunity to gain insights into this particular pathway and the structure–function relationship of this novel group of enzymes.

## Materials and methods

### Data mining for CGTases

Metagenomes were analyzed from the Joint Genome Institute (JGI) IMG/M database^103^, which contains more than 15,014 metagenomes from different environments (last search, July 2021). Putative CGTases were detected by a BLASTn search in ~ 130 publicly assembled metagenomes in the IMG/M platform using an *E*-value cutoff of 1.0e^−5^. The metagenomes were filtered for those containing different terms from hyperthermophilic ecological niches (*e.g.*, geothermal fumarole, geyser, hot spring, or hydrothermal vent) in the "Genome Name/Sample Name" description (Table S1). The protein query sequences consisted of the complete amino acid sequences of experimentally characterized CGTases, including CGTase from G+ *T. thermosulfurigenes* EM1 with a conventional five-domain ABCDE_CBM20_ distribution (NCBI ID: AAB00845.1, PDB ID: 1CIU)^21^, the solely characterized CGTase from G− *K. oxytoca* M5a1 with a four-domain ABCE_CBM20_ distribution (NCBI ID: AAA25059.1)^104^, and a CGTase from the thermophilic archaea *P. furiosus* DSM 3638 with a five-domain ABCDE_arch_ distribution (NCBI ID: ABA33720.1)^105^. Putative CGTases that shared > 45% sequence identity with query sequences were excluded to increase novelty. The best hits were analyzed manually to evaluate the complete scaffold templates and discard truncated sequences. NCBI's Batch Web CD-Search Tool against the Conserved Domain Database (CDD/SPARCLE)^106^ was employed to predict the functional domains of selected hits. Hence, a putative CGTase with a unique three-domain ABC distribution (named CldA) was identified in a scaffold containing ~ 50 genes in a metagenome of thermophilic microbial communities from Obsidian Pool hot spring at Yellowstone National Park (Wyoming, USA) (Table S1). Therefore, a second database mining approach was applied to identify additional three-domain ABC CGTases. The CldA sequence was then submitted to BLASTn against 30 publicly assembled metagenomes deposited in the IMG/M platform^103^ that belong to several microbial communities from the Obsidian Pool hot spring at Yellowstone National Park (Table S2). A second putative three-domain ABC CGTase (named ThmA) was identified in three metagenomes from the Obsidian Pool hot spring (Table S2). Redundant sequences and truncated genes were discarded. The CldA/ThmA sequences, along with the 51 sequences of characterized enzymes from GH13_2, were listed into a FASTA file and subjected to multiple alignments using Clustal Omega with default parameters^107^. Manual refinement of the multiple alignments was performed to detect key conserved catalytic residues from CGTases^13,31,62^. Finally, a third database mining approach was conducted to identify additional (CldA/ThmA)-like CGTases. Hence, the CldA/ThmA sequences were submitted to BLASTn against publicly assembled genomes deposited in the GenBank database from *Caldanaerobacter* spp. (NCBI Taxonomy ID: 249529) and *Thermoanaerobacter* spp. (NCBI Taxonomy ID: 68295). Several (CldA/ThmA)-like sequences were obtained (Table S3), listed in a FASTA file, and subjected to the bioinformatics pipeline described above. The sequence logo was generated by WebLogo^108^. The three-domain ABC CGTase CldA was selected for further recombinant production and functional studies.

### Gene cloning and protein production

A synthetic gene coding for the mature form of CldA, codon-optimized for *E. coli* expression, was prepared by Integrated DNA Technologies (Iowa, USA). The synthetic *cldA* gene was cloned into the NdeI and NotI sites of the pET-22b(+) expression vector (Novagen), which contains a sequence coding for six histidines at the C-terminus. The identity of the resulting plasmid pCldA was evaluated by restriction analysis and confirmed by DNA sequencing. Electrocompetent *E. coli* BL21(DE3)pLysS cells were transformed with pCldA and grown on Luria–Bertani (LB) agar plates containing 100 μg mL^−1^ ampicillin at 37 °C. A single colony of BL21(DE3)pLysS/pCldA was picked to inoculate 5 mL LB medium overnight with 100 μg mL^−1^ ampicillin at 37 °C, aliquoted in a sterile solution of 40% (*v/v*) glycerol and maintained at − 80 °C. For recombinant CldA production, a fraction of a frozen cell aliquot was taken and cultured for 12 h at 37 °C and 200 rev min^−1^ in 50 mL LB medium containing 200 μg mL^−1^ ampicillin. This preinoculum was used to inoculate 1 L 2xYT medium with 200 μg mL^−1^ ampicillin at an initial optical density at 600 nm (OD_600_) of 0.05 at 37 °C and 200 rev min^−1^. After induction by adding a final concentration of 0.1 mM IPTG to the medium (OD_600_ of ~ 0.6), the temperature was lowered to 22 °C, and the culture was grown for 12 h at 150 rev min^−1^. The cells were harvested by centrifugation (7500*g*, 10 min, 4 °C) and resuspended in 10 mL buffer A [50 mM sodium phosphate pH 8.0, 500 mM NaCl, 2% (*v/v*) glycerol, 20 mM imidazole] containing EDTA-free complete protease inhibitor cocktail mini tablet (Roche Molecular Biochemicals) and 1 μg mL^−1^ DNAse. The cell suspension was sonicated on ice for 30 min with an amplitude of 25–29%, and the resulting solution was heated for 20 min at 60 °C to precipitate the thermolabile protein fraction of *E. coli*. After the heating step, the lysate was centrifuged (19,000*g*, 45 min, 4 °C), and the supernatant containing recombinant thermophilic His_6_-tagged CldA was recovered.

### Protein purification and SEC-DLS analysis

The supernatant containing CldA was filtered with a 0.22 μm pore filter and applied onto a 5 mL Ni^2+^-chelating HisTrap HP column (GE Healthcare) equilibrated with ten-bed volumes of buffer A using an ÄKTA Pure 25 M1 FPLC system with UNICORN software (GE Healthcare). The column was then washed with eight-bed volumes of buffer A to remove contaminants. Bound CldA enzyme was eluted with a linear gradient of 20–500 mM imidazole using buffer B [50 mM sodium phosphate pH 8.0, 500 mM NaCl, 2% (*v/v*) glycerol, 500 mM imidazole] at a flow rate of 5 mL min^−1^ and analyzed by SDS-PAGE with Coomassie staining. A single peak at ~ 300 mM imidazole containing the CldA enzyme was collected, concentrated, and dialyzed against several volumes of buffer C [50 mM Tris–HCl pH 7.5, 100 mM NaCl] in an ultrafiltration cell (Amicon Ultracel filter, 30 kDa molecular-weight cutoff). The SEC-DLS analysis was performed using an ÄKTA Pure 25 M1 FPLC system coupled to a dynamic light scattering (DLS) detector using a Malvern Zetasizer μV DLS instrument. A concentrated sample of CldA at 10 mg mL^−1^ was filtrated with a 0.22 μm pore filter and loaded onto a 120 mL HiLoad 16/600 Superdex 75 pg column (GE Healthcare) equilibrated with buffer C. CldA was then eluted with the same buffer in an SEC-DLS coupled experiment using a quartz flow cell of 8 μL (Malvern) at a flow rate of 1.0 mL min^−1^. The SEC-DLS system was previously calibrated with a standard of bovine serum albumin (BSA, Sigma-Aldrich) at 17 mg mL^−1^ in buffer C. Data acquisition and analysis of SEC-DLS measurements were carried out using the OmniSEC 5.12 software (Malvern). A highly purified and monodisperse peak corresponding to the CldA monomer (58.5 kDa) was collected, concentrated, and dialyzed against several volumes of buffer D [50 mM Tris–HCl pH 7.5] using a 30 kDa cutoff ultrafiltration cell for enzyme activity assays. Protein concentration was determined by the Bradford assay using BSA as a standard.

### Enzyme activity assay

The reaction mixture (1 mL) at 75 °C consisted of 50 mM sodium acetate pH 4.0, 5% (*w/v*) soluble starch (Sigma-Aldrich, Product Number: S9765), 10 mM CaCl_2_, and 1 µg (1.71e^−5^ μmol) purified CldA enzyme. The initial rates were measured using a 96-well microplate reader (Multiskan Sky Microplate Spectrophotometer, Thermo Fisher Scientific). The temperature dependence of β-CGTase activity was determined in the 40–100 °C range. The optimum pH was determined by incubating the enzyme in different 50 mM buffer solutions ranging from pH 3.0 to 9.0. Hence, glycine–HCl buffer was used at pH 3.0, acetate buffer at pH 4.0 to 5.0, phosphate buffer at pH 6.0 to 7.0, Tris–HCl buffer at pH 8.0, and glycine–NaOH buffer at pH 9.0. The β-CGTase activity was determined spectrophotometrically by the phenolphthalein method described elsewhere^109^ with minor modifications. Accordingly, 250 mL of working phenolphthalein solution was prepared by adding ~ 249 mL of 125 mM sodium carbonate pH 10.5 to 1 mL of 3 mM phenolphthalein solution in ethanol. The reaction was stopped by adding 175 µL of 1 mM NaOH to 50 µL aliquots of the reaction mixture. The latter solution was then mixed and vortexed with 100 µL of working phenolphthalein solution and analyzed by the decrease in absorbance at λ = 550 nm owing to β-CD-phenolphthalein complex formation. The β-CD concentration was determined using a standard curve constructed by the phenolphthalein method^109^ with commercial β-CD (Sigma-Aldrich). One unit of β-CGTase activity was defined as the amount of enzyme that produced 1 μmol β-CD per min under the defined conditions. The hydrolytic activity was measured as the liberation of reducing sugars from soluble starch by the 3,5-dinitrosalicylic acid (DNS) method^110^ using a standard curve constructed with commercial maltose (Sigma-Aldrich). One unit of hydrolytic activity was defined as the amount of enzyme that produced 1 μmol of reducing sugars per min under the defined conditions.

### Product analysis

The formation of CDs from starch was determined by incubating 1.71e^−2^ μM CldA with 5% (*w/v*) soluble starch in 50 mM sodium acetate pH 4.0 and 10 mM CaCl_2_ at 75 °C for 4 h. Aliquots of 1 mL were taken at regular intervals, and the reactions were stopped by adding two volumes of cold HPLC water. The mixtures were centrifuged (16,000*g*, 15 min) and filtered through a 0.22 μm polyvinylidene difluoride (PDVF) membrane filter (GVS Life Sciences). Products formed were analyzed using a Waters Alliance HPLC system (Model e2695 Separations Module, USA) employing a Waters XBridge BEH amide column (5 µm, 150 mm × 4.6 mm) heated at 30 °C. Samples were processed at an eluent of acetonitrile:water (65:35, *v/v*) with a flow rate of 0.5 mL min^−1^ using a refractive index (RI) detector (Model 2414, Waters) also heated at 30 °C. Data acquisition and treatment were performed with Empower software v.2.0 (Waters). Mass spectrometry analysis of products from 5% (w/v) soluble starch by the action of CldA was obtained from a mixture at 2 h using a QTOF Xevo G2-S (Waters). A direct infusion into the mass spectrometer was used at a flow rate of 5 μL min^−1^. The ionization conditions were as follows: (i) the electrospray source was operated in positive ion mode, and the source and desolvation temperatures were 100 and 250 °C, respectively; (ii) desolvation and cone gas at a flow rate of 800 and 50 L h^−1^, respectively; (iii) capillary and cone voltage of 2500 and 10 V, respectively; (iv) acquisition mass range from 50 to 1500 m/z. For HPLC and mass spectrometry analysis, high-purity oligosaccharides from G3 to G7 (Toronto Research Chemical) and G1-G2, α-, β-, and γ-CDs (Sigma-Aldrich) were used as standards.

### Phylogenetic analyses

The phylogenetic tree was based on the alignment of 78 amino acid sequences of several CGTases, including the 48 characterized CGTases from GH13_2 recognized in the CAZy database, 19 three-domain ABC (CldA/ThmA)-like CGTases, and 11 putative CGTases (NCBI ID: SPM25085.1, WP_102522592.1, WP_077677735.1, WP_013906564.1, WP_048149607.1, WP_048164181.1, WP_071127407.1, WP_115251089.1, WP_078681113.1, WP_069649891.1, and WP_069657150.1) to improve the fit of some clades (Fig. 3). The sequences of 7 α-amylases from GH13 were used as an outgroup. Three starch-acting enzymes from GH13_2 (NCBI ID: AAA22229.1, AID53183.1, and CAJ81031.1) were excluded from the analysis since they are not CGTases. Two phylogenetic trees were built using the full amino acid sequence (Fig. 3) or solely the amino acid sequence of the minimal functional core ABC (Fig. S6) for all 85 sequences mentioned above. The alignment of all amino acid sequences was conducted with the ClustalW algorithm using default parameters. The evolutionary relationship of CGTases was inferred with the maximum likelihood method^111^, setting the best-fit model of amino acid substitution (WAG + G)^112^. The bootstrap method (1000 replicates) was applied to assess the confidence in the phylogenetic analysis. All the implemented algorithms are included in the Molecular Evolutionary Genetics Analysis (MEGA 6.06) package^112^. The consensus tree was visualized and edited in Interactive Tree Of Life iTOL v4 (http://itol.embl.de)^113^.

### Data mining for CM-CD gene clusters

The *cld* gene clusters where the *cld*A-like-encoding genes are located were delimited in the complete assembled scaffold from *C. subterraneus* ssp. *yonseiensis* KB-1 (NCBI ID: AXDC01000002, location 50928–86345), *C. subterraneus* ssp. *subterraneus* 38_43 (NCBI ID: LGEY01000002, location 21575–56994) and *T. tengcongensis* MB4 (NCBI ID: AE008691.1, location 1749287–1786305). Partial scaffolds of the *cld* gene clusters were also found in five other genomes from *C. subterraneus* ssp. (Tables S3, S4). Furthermore, the *cld* gene clusters involved in the CM-CD pathway were submitted to BLASTn against 246 available genomes deposited in the GenBank database from Thermoanaerobacterales order (NCBI Taxonomy ID: 68295). Accordingly, the genomes from *Carboxydothermus* (NCBI Taxonomy ID: 129957), *Thermacetogenium* (NCBI Taxonomy ID: 140458), *Gelria* (NCBI Taxonomy ID: 189326), *Desulfovirgula* (NCBI Taxonomy ID: 418453), *Tepidanaerobacter* (NCBI Taxonomy ID: 499228), *Fervidicola* (NCBI Taxonomy ID: 555078), *Caldanaerobius* (NCBI Taxonomy ID: 862261), *Brockia* (NCBI Taxonomy ID: 1648500), *Calorimonas* (NCBI Taxonomy ID: 2606906), *Thermodesulfitimonas* (NCBI Taxonomy ID: 1914252), and *Moorella* (NCBI Taxonomy ID: 44260), as well as Thermodesulfobiaceae *Thermodesulfobium* (NCBI Taxonomy ID: 227388), Thermoanaerobacterales family III *Anaerocellum* (NCBI Taxonomy ID: 33955), *Caldicellulosiruptor* (NCBI Taxonomy ID: 44000), *Thermovenabulum* (NCBI Taxonomy ID: 159730), *Thermosediminibacter* (NCBI Taxonomy ID: 291988), *Caldanaerovirga* (NCBI Taxonomy ID: 591374), *Syntrophaceticus* (NCBI Taxonomy ID: 862071), and Thermoanaerobacterales family IV *Mahella* (NCBI Taxonomy ID: 252965) were analyzed for CM-CD gene clusters. Similarly, both *thm* and *thb* gene clusters were detected using an expanded searching cross-families algorithm in the Pathosystems Resource Integration Center (PATRIC v.3.6.8) database^114^. Hence, the *thm* gene cluster was detected in the complete assembled scaffold of several species from *Thermoanaerobacter*, including *Thermoanaerobacter pseudethanolicus* ATCC 33223 (NCBI ID: CP000924), *Thermoanaerobacter indiensis* BSB-33 (NCBI ID: ARDJ00000000), *Thermoanaerobacter brockii* ssp. *finnii* Ako-1 (NCBI ID: CP002466), and *Thermoanaerobacter* sp. strains X513 (NCBI ID: CP002210), X514 (NCBI ID: CP000923), UBA8867 (NCBI ID: DOPY00000000), and X561 (NCBI ID: ACXP00000000). Meanwhile, the *thb* gene cluster was detected in the complete assembled scaffold from *Thermoanaerobacterium aotearoense* SCUT27 (NCBI ID: AYSN00000000), *Thermoanaerobacterium saccharolyticum* JW/SL-YS485 (NCBI ID: CP003184), *Thermoanaerobacterium xylanolyticum* LX-11 (NCBI ID: CP002739), and *Thermoanaerobacterium thermosaccharolyticum* DSM 571 (NCBI ID: CP002171). Functional comparisons of the *cld*, *thm,* and *thb* gene clusters with the *cym, cgt,* and *cyc* gene clusters involved in the CM-CD pathway from *K. oxytoca* M5a1 (NCBI ID: CP020657; location 664764–678182), *Thermococcus* sp. B1001 (NCBI ID: AB034969.2) and *B. subtilis* (NCBI ID: CP011534; location 3355114–3365346), respectively, were performed using the PATRIC genus-specific protein families (PLFams) method^114^. Functional prediction of proteins encoded by the *cld*, *thm,* and *thb* gene clusters (Table S4) was carried out using the CDD/SPARCLE^106^, Pfam^115^, and UniProt (https://www.uniprot.org/) databases. Protein subcellular localization and physicochemical property predictions were conducted using the CELLO v.2.5^116^ and ProtParam (ExPASy) servers^117^, respectively. The presence of a signal peptide was performed using the SignalP 5.0 server^118^. Metabolic pathway analysis was conducted using the Kyoto Encyclopedia of Genes and Genomes (KEGG) database^119^.

## Supplementary Information

Supplementary Figures.

Supplementary Table S1.

Supplementary Table S2.

Supplementary Table S3.

Supplementary Table S4.

## References

[1] Crini G (2014). Review: A history of cyclodextrins. Chem. Rev..

[2] Jambhekar SS, Breen P (2016). Cyclodextrins in pharmaceutical formulations I: Structure and physicochemical properties, formation of complexes, and types of complex. Drug Discov. Today.

[3] Strompen S, Miranda-Molina A, López-Munguía A, Castillo E, Saab-Rincón G (2015). Acceptor-induced modification of regioselectivity in CGTase-catalyzed glycosylations of *p*-nitrophenyl-glucopyranosides. Carbohydr. Res..

[4] Jones ST (2020). Modified cyclodextrins as broad-spectrum antivirals. Sci. Adv..

[5] Fan J, Zang Y, Jiang J, Lei J, Xue H (2019). Beta-cyclodextrin-functionalized CdS nanorods as bulding modules for ultrasensitive photoelectrochemical bioassay of HIV DNA. Biosens. Bioelectron..

[6] Mousset E (2014). Influence of solubilizing agents (cyclodextrin or surfactant) on phenanthrene degradation by electro-fenton process—Study of soil washing recycling possibilities and environmental impact. Water Res..

[7] Yu G, Jie K, Huang F (2015). Supramolecular amphiphiles based on host-guest molecular recognition motifs. Chem. Rev..

[8] Shishido TK (2015). Antifungal activity improved by coproduction of cyclodextrins and anabaenolysins in Cyanobacteria. Proc. Natl. Acad. Sci. USA..

[9] Wen Y (2020). A supramolecular platform for controlling and optimizing molecular architectures of siRNA targeted delivery vehicles. Sci. Adv..

[10] Bardi L, Mattei A, Steffan S, Marzona M (2000). Hydrocarbon degradation by a soil microbial population with β-cyclodextrin as surfactant to enhance bioavailability. Enzyme Microb. Technol..

[11] Trotta F, Zanetti M, Camino G (2000). Thermal degradation of cyclodextrins. Polym. Degrad. Stab..

[12] Kohata S, Jyodoi K, Ohyoshi A (1992). Thermal decomposition of cyclodextrins (α-, β-, γ-, and modified β-CyD) and of metal—(β-CyD) complexes in the solid phase. Thermochim. Acta.

[13] Uitdehaag JC, Kalk KH, van der Veen BA, Dijkhuizen L, Dijkstra BW (1999). The cyclization mechanism of cyclodextrin glycosyltransferase (CGTase) as revealed by a gamma-cyclodextrin-CGTase complex at 1.8-Å resolution. J. Biol. Chem..

[14] Lombard V, Golaconda Ramulu H, Drula E, Coutinho PM, Henrissat B (2014). The carbohydrate-active enzymes database (CAZy) in 2013. Nucleic Acids Res..

[15] Stam MR, Danchin EGJ, Rancurel C, Coutinho PM, Henrissat B (2006). Dividing the large glycoside hydrolase family 13 into subfamilies: Towards improved functional annotations of α-amylase-related proteins. Protein Eng. Des. Sel..

[16] Janecek S, Svensson B, MacGregor EA (2014). α-Amylase: An enzyme specificity found in various families of glycoside hydrolases. Cell. Mol. Life Sci..

[17] Meng X (2016). Structure–function relationships of family GH70 glucansucrase and 4,6-α-glucanotransferase enzymes, and their evolutionary relationships with family GH13 enzymes. Cell. Mol. Life Sci..

[18] Uitdehaag JC (1999). X-ray structures along the reaction pathway of cyclodextrin glycosyltransferase elucidate catalysis in the α-amylase family. Nat. Struct. Biol..

[19] Janecek S, MacGregor EA, Svensson B (1995). Characteristic differences in the primary structure allow discrimination of cyclodextrin glucanotransferases from alpha-amylases. Biochem. J..

[20] van der Veen BA, van Alebeek GJ, Uitdehaag JC, Dijkstra BW, Dijkhuizen L (2000). The three transglycosylation reactions catalyzed by cyclodextrin glycosyltransferase from *Bacillus circulans* (strain 251) proceed via different kinetic mechanisms. Eur. J. Biochem..

[21] Knegtel RMA, Wind D, Dijkstra BW (1996). Crystal structure at 2.3 Å resolution and revised nucleotide sequence of the thermostable cyclodextrin glycosyltransferase from *Thermoanaerobacterium thermosulfurigenes* EM1. J. Mol. Biol..

[22] Penninga D (1996). The raw starch binding domain of cyclodextrin glycosyltransferase from *Bacillus circulans* strain 251. J. Biol. Chem..

[23] Janeček Š, Mareček F, MacGregor EA, Svensson B (2019). Starch-binding domains as CBM families-history, occurrence, structure, function and evolution. Biotechnol. Adv..

[24] Christiansen C (2009). The carbohydrate-binding module family 20—Diversity, structure, and function. FEBS J..

[25] Rimphanitchayakit V, Tonozuka T, Sakano Y (2005). Construction of chimeric cyclodextrin glucanotransferases from *Bacillus circulans* A11 and *Paenibacillus macerans* IAM1243 and analysis of their product specificity. Carbohydr. Res..

[26] Ohdan K, Kuriki T, Takata H, Kaneko H, Okada S (2000). Introduction of raw starch-binding domains into *Bacillus subtilis* α-amylase by fusion with the starch-binding domain of *Bacillus* cyclomaltodextrin glucanotransferase. Appl. Environ. Microbiol..

[27] Strokopytov B (1996). Structure of cyclodextrin glycosyltransferase complexed with a maltononaose inhibitor at 2.6 Å resolution. Implications for product specificity. Biochemistry.

[28] van der Veen BA, Uitdehaag JCM, Dijkstra BW, Dijkhuizen L (2000). Engineering of cyclodextrin glycosyltransferase reaction and product specificity. Biochem. Biophys. Acta..

[29] van der Veen BA (2001). Hydrophobic amino acid residues in the acceptor binding site are main determinants for reaction mechanism and specificity of cyclodextrin-glycosyltransferase. J. Biol. Chem..

[30] Leemhuis H, Dijkstra B, Dijkhuizen L (2002). Mutations converting cyclodextrin glycosyltransferase from a transglycosylase into a starch hydrolase. FEBS Lett..

[31] Yamamoto T (2000). Alteration of product specificity of cyclodextrin glucanotransferase from *Thermococcus* sp. BlOOl by site-directed mutagenesis. J. Biosci. Bioeng..

[32] Kelly RM, Leemhuis H, Dijkhuizen L (2007). Conversion of a cyclodextrin glucanotransferase into an α-amylase: Assessment of directed evolution strategies. Biochemistry.

[33] Kelly RM (2008). Elimination of competing hydrolysis and coupling side reactions of a cyclodextrin glucanotransferase by directed evolution. Biochem. J..

[34] Wang L, Duan X, Wu J (2016). Enhancing the α-cyclodextrin specificity of cyclodextrin glycosyltransferase from *Paenibacillus macerans* by mutagenesis masking subsite -7. Appl. Environ. Microbiol..

[35] Janeček Š, Svensson B, MacGregor EA (2003). Relation between domain evolution, specificity, and taxonomy of the α-amylase family members containing a C-terminal starch-binding domain. Eur. J. Biochem..

[36] Rashid N (2002). Characterization of an archaeal cyclodextrin glucanotransferase with a novel C-terminal domain. J. Bacteriol..

[37] Fiedler G, Pajatsch M, Böck A (1996). Genetics of a novel starch utilisation pathway present in *Klebsiella oxytoca*. J. Mol. Biol..

[38] Labes A, Schönheit P (2007). Unusual starch degradation pathway via cyclodextrins in the hyperthermophilic sulfate-reducing archaeon *Archaeoglobus fulgidus* strain 7324. J. Bacteriol..

[39] Oslowski DM, Jung JH, Seo DH, Park CS, Holden JF (2011). Production of hydrogen from α-1,4- and β-1,4-linked saccharides by marine hyperthermophilic Archaea. Appl. Environ. Microbiol..

[40] Pajatsch M (1998). The periplasmic cyclodextrin binding protein CymE from *Klebsiella oxytoca* and its role in maltodextrin and cyclodextrin transport. J. Bacteriol..

[41] Kamionka A, Dahl MK (2001). *Bacillus subtili*s contains a cyclodextrin-binding protein which is part of a putative ABC-transporter. FEMS Microbiol. Lett..

[42] Shim JH (2009). Role of maltogenic amylase and pullulanase in maltodextrin and glycogen metabolism of *Bacillus subtilis* 168. J. Bacteriol..

[43] Van Den Berg B, Bhamidimarri SP, Prajapati JD, Kleinekathöfer U, Winterhalter M (2015). Outer-membrane translocation of bulky small molecules by passive diffusion. Proc. Natl. Acad. Sci. USA..

[44] Thomas C, Tampé R (2020). Structural and mechanistic principles of ABC transporters. Annu. Rev. Biochem..

[45] Hashimoto Y, Yamamoto T, Fujiwara S, Takagi M, Imanaka T (2001). Extracellular synthesis, specific recognition, and intracellular degradation of cyclomaltodextrins by the hyperthermophilic archaeon *Thermococcus* sp. strain B1001. J. Bacteriol..

[46] Hulsmann A, Lurz R, Scheffel F, Schneider E (2000). Maltose and maltodextrin transport in the thermoacidophilic gram-positive bacterium *Alicyclobacillus acidocaldarius* is mediated by a high-affinity transport system that includes a maltose binding protein tolerant to low pH. J. Bacteriol..

[47] Tonozuka T (2007). Structural basis for cyclodextrin recognition by *Thermoactinomyces vulgaris* cyclo/maltodextrin-binding protein. FEBS J..

[48] Leisico F (2020). Multitask ATPases (NBDs) of bacterial ABC importers type I and their interspecies exchangeability. Sci. Rep..

[49] Zeldes BM (2015). Extremely thermophilic microorganisms as metabolic engineering platforms for production of fuels and industrial chemicals. Front. Microbiol..

[50] Roth C (2017). Amylose recognition and ring-size determination of amylomaltase. Sci. Adv..

[51] Hemme CL (2011). Correlation of genomic and physiological traits of *Thermoanaerobacter* species with biofuel yields. Appl. Environ. Microbiol..

[52] Jørgensen ST, Tangney M, Starnes RL, Amemiya K, Jørgensen PL (1997). Cloning and nucleotide sequence of a thermostable cyclodextrin glycosyltransferase gene from *Thermoanaerobacter* sp. ATCC 53627 and its expression in *Escherichia coli*. Biotechnol. Lett..

[53] Ara KZG (2015). A CGTase with high coupling activity using γ-cyclodextrin isolated from a novel strain clustering under the genus *Carboxydocella*. Glycobiology.

[54] Wind LED (1995). Cyclodextrin formation by the thermostable α-amylase of *Thermoanaerobacterium thermosulfurigenes* EM1 and reclassification of the enzyme as a cyclodextrin glycosyltransferase. Appl. Environ. Microbiol..

[55] Sant Anna FH, Lebedinsky AV, Sokolova TG, Robb FT, Gonzalez JM (2015). Analysis of three genomes within the thermophilic bacterial species *Caldanaerobacter subterraneus* with a focus on carbon monoxide dehydrogenase evolution and hydrolase diversity. BMC Genomics.

[56] Lee S-J (2013). Draft genome sequence of an anaerobic and extremophilic bacterium, *Caldanaerobacter yonseiensis*, isolated from a geothermal hot stream. Genome Announc..

[57] Martinez-Alonso E (2019). Taxonomic and functional characterization of a microbial community from a volcanic englacial ecosystem in Deception Island, Antarctica. Sci. Rep..

[58] Vishnivetskaya TA (2014). Community analysis of plant biomass-degrading microorganisms from Obsidian Pool, Yellowstone National Park. Microb. Ecol..

[59] Tjalsma H (1999). The role of lipoprotein processing by signal peptidase II in the Gram-positive Eubacterium *Bacillus subtilis*. J. Biol. Chem..

[60] Yan S, Wu G (2017). Bottleneck in secretion of α-amylase in *Bacillus subtilis*. Microb. Cell Fact..

[61] Schneenwind O, Missiakas D (2015). Sec-secretion and sortase-mediated anchoring of proteins in Gram-postive Bacteria. Biochim. Biophys. Acta.

[62] Xie T, Song B, Yue Y, Chao Y, Qian S (2014). Site-saturation mutagenesis of central tyrosine 195 leading to diverse product specificities of an α-cyclodextrin glycosyltransferase from *Paenibacillus* sp. 602-1. J. Biotechnol..

[63] Beier L (2000). Conversion of the maltogenic α-amylase Novamyl into a CGTase. Protein Eng..

[64] Dauter Z (1999). X-ray structure of Novamyl, the five-domain “maltogenic” α-amylase from *Bacillus stearothermophilus*: Maltose and acarbose complexes at 1.7 Å resolution. Biochemistry.

[65] Fardeau M (2004). Isolation from oil reservoirs of novel thermophilic anaerobes phylogenetically related to *Thermoanaerobacter subterraneus*: Reassignment of *T. subterraneus*, *Thermoanaerobacter yonseiensis*, *Thermoanaerobacter tengcongensis* and *Carboxydibrachium pacificum*. Int. J. Syst. Evol. Microbiol..

[66] Korzhenkov AA, Toshchakov SV, Podosokorskaya OA, Patrushev MV, Kublanov IV (2020). Data on draft genome sequence of *Caldanaerobacter* sp. strain 1523vc, a thermophilic bacterium, isolated from a hot spring of Uzon Caldera, (Kamchatka, Russia ). Data Brief.

[67] Desvaux M, Dumas E, Chafsey I, Hébraud M (2006). Protein cell surface display in Gram-positive bacteria: From single protein to macromolecular protein structure. FEMS Microbiol. Lett..

[68] Podkovyrov SM, Zeikus JG (1992). Structure of the gene encoding cyclomaltodextrinase from *Clostridium thermohydrosulfuricum* 39E and characterization of the enzyme purified from *Escherichia coli*. J. Bacteriol..

[69] Zheng Y (2010). Cloning, expression, and characterization of a thermostable glucoamylase from *Thermoanaerobacter tengcongensis* MB4. Appl. Microbiol. Biotechnol..

[70] Chen S (2007). Molecular investigation of a novel thermostable glucan phosphorylase from *Thermoanaerobacter tengcongensis*. Enzyme Microb. Technol..

[71] Zhou C-Q, Wang J-Q, Qian Z, Ma Y-H, Liu S-Q (2006). Cloning, expression and characterization of the 6-phosphofructokinase from *Thermoanaerobacter tengcongensis*. Wei Sheng Wu Xue Bao.

[72] Navdaeva V (2011). Phosphoenolpyruvate: Sugar phosphotransferase system from the hyperthermophilic *Thermoanaerobacter tengongensis*. Biochemistry.

[73] Lin L (2011). The *Thermoanaerobacter* glycobiome reveals mechanisms of pentose and hexose co-utilization in bacteria. PLoS Genet..

[74] Zhu M, Lu Y, Wang J, Li S, Wang X (2015). Carbon catabolite repression and the related genes of *ccp*A, *pts*H and *hpr*K in *Thermoanaerobacterium aotearoense*. PLoS ONE.

[75] McHenry CS (2011). Bacterial replicases and related polymerases. Curr. Opin. Chem. Biol..

[76] Anashkin VA (2015). Cystathionine Beta Synthase (CBS) domain-containing pyrophosphatase as a target for diadenosine polyphosphates in bacteria. J. Biol. Chem..

[77] Baykov AA, Tuominen HK, Lahti R (2011). The CBS Domain : A Protein module with an emerging prominent role in regulation. ACS Chem. Biol..

[78] Vemula H, Ayon NJ, Gutheil WG (2015). Cytoplasmic peptidoglycan intermediate levels in *Staphylococcus aureus*. Biochimie.

[79] Geno KA, Hauser JR, Gupta K, Yother J (2014). *Streptococcus pneumoniae* phosphotyrosine phosphatase CpsB and availability. J. Bacteriol..

[80] Gonzalez GM (2017). Structural insights into RapZ-mediated regulation of bacterial amino-sugar metabolism. Nucleic Acids Res..

[81] Muchová K, Chromiková Z, Valencíková R, Barák I (2018). Interaction of the morphogenic protein RodZ with the *Bacillus subtilis* Min System. Front. Microbiol..

[82] Stamsas GA (2017). Identification of EloR (Spr1851) as a regulator of cell elongation in *Streptococcus pneumoniae*. Mol. Microbiol..

[83] Kaiser BK, Clifton MC, Shen BW, Stoddard BL (2009). The structure of a bacterial DUF199/WhiA protein: Domestication of an invasive endonuclease. Cell Struct..

[84] Ainsa JA, Ryding NJ, Hartley N, Findlay KC, Bruton CJ, Chater KF (2000). WhiA, a protein of unknown function conserved among Gram-positive bacteria, is essential for sporulation in *Streptomyces coelicolor* A3 (2). J. Bacteriol..

[85] Abokitse K, Wu M, Bergeron H, Grosse S, Lau PCK (2010). Thermostable feruloyl esterase for the bioproduction of ferulic acid from triticale bran. Appl. Microbiol. Biotechnol..

[86] Oyugi MA, Bashiri G, Baker EN, Johnson-winters KL (2016). Investigating the reaction mechanism of F420-dependent glucose-6-phosphate dehydrogenase from *Mycobacterium tuberculosis*: Kinetic analysis of the wild-type and mutant enzymes. Biochemistry.

[87] Greening C (2016). Physiology, biochemistry, and applications of F420- and Fo-dependent redox reactions. Microb. Mol. Biol. Rev..

[88] Hans M (1999). 2-Hydroxyglutaryl-CoA dehydratase from *Clostridium symbiosum*. Eur. J. Biochem. FEBS.

[89] Tillander V, Alexson SEH, Cohen DE (2018). Deactivating fatty acids: Acyl-CoA thioesterase-mediated control of lipid metabolism. Trends Endocrinol. Metab..

[90] Arcondeguy T, Jack R, Merrick M (2001). P II Signal transduction proteins, pivotal players in microbial nitrogen control. Microbiol. Mol. Biol. Rev..

[91] Badger J (2005). Structural analysis of a set of proteins resulting from a bacterial genomics project. Proteins.

[92] Johansson MJO, Byström AS (2002). Dual function of the tRNA (m5U54) methyltransferase in tRNA maturation. RNA.

[93] He X (2011). Structure of a cation-bound multidrug and toxic compound extrusion transporter. Nature.

[94] Hvorup RN (2003). The multidrug/oligosaccharidyl-lipid/polysaccharide (MOP) exporter superfamily. Eur. J. Biochem..

[95] Del-Rio G, Morett E, Soberon X (1997). Did cyclodextrin glycosyltransferases evolve from α-amylases?. FEBS Lett..

[96] Kuchtová A, Gentry MS, Janecek S (2018). The unique evolution of the carbohydrate-binding module CBM20 in laforin. FEBS Lett..

[97] Ngo ST (2019). Interaction of carbohydrate binding module 20 with starch substrates. RSC Adv..

[98] Ludwig W, Schleifer K-H, Whitman W (2009). Bergey’s Manual of Systematic Bacteriology.

[99] Ferreira MJ, De Sá-Nogueira I (2010). A multitask ATPase serving different ABC-type sugar importers in *Bacillus subtilis*. J. Bacteriol..

[100] Schönert S (2006). Maltose and maltodextrin utilization by *Bacillus subtilis*. J. Bacteriol..

[101] Sahm K, Matuschek M, Müller H, Mitchell WJ, Bahl H (1996). Molecular analysis of the *amy* gene locus of *Thermoanaerobacterium thermosulfurigenes* EM1 encoding starch-degrading enzymes and a binding protein-dependent maltose transport system. J. Bacteriol..

[102] Matuschek M, Burchhardt G, Sahm K, Bahl H (1994). Pullulanase of *Thermoanaerobacterium thermosulfurigenes* EM1 (*Clostridium thermosulfurogenes*): Molecular analysis of the gene, composite structure of the enzyme, and a common model for its attachment to the cell surface. J. Bacteriol..

[103] Chen IA (2019). IMG/M v.5.0: An integrated data management and comparative analysis system for microbial genomes and microbiomes. Nucleic Acids Res..

[104] Binder F, Huberb O, August B (1986). Cyclodextrin-glycosyltransferase from *Klebsiella pneumoniue* M5al: Cloning, nucleotide sequence and expression. Gene.

[105] Lee MH (2007). Characterization of a thermostable cyclodextrin glucanotransferase from *Pyrococcus furiosus* DSM3638. Extremophiles.

[106] Marchler-Bauer A (2017). CDD/SPARCLE: Functional classification of proteins via subfamily domain architectures. Nucleic Acids Res..

[107] Sievers F, Higgins DG (2018). Clustal Omega for making accurate alignments of many protein sequences. Protein Sci..

[108] Crooks GE, Hon G, Chandonia J, Brenner SE (2004). WebLogo: A sequence logo generator. Genome Res..

[109] Goel A, Nene S (1995). A novel cyclomaltodextrin glucanotransferase from *Bacillus firmus* that degrades raw starch. Biotechnol. Lett..

[110] Miller GL (1959). Use of dinitrosaIicyIic acid reagent for determination of reducing sugar. Anal. Chem..

[111] Milligan BG (2003). Maximum-likelihood estimation of relatedness. Genetics.

[112] Tamura K, Stecher G, Peterson D, Filipski A, Kumar S (2013). MEGA6: Molecular evolutionary genetics analysis version 6.0. Mol. Biol. Evol. Soc..

[113] Letunic I, Bork P (2019). Interactive Tree Of Life (iTOL) v4: Recent updates and. Nucleic Acids Res..

[114] Davis JJ (2020). The PATRIC Bioinformatics Resource Center: Expanding data and analysis capabilities. Nucleic Acids Res..

[115] Finn RD (2014). Pfam: The protein families database. Nucleic Acids Res..

[116] Yu C-S, Chen Y-C, Lu C-H, Hwang J-K (2006). Prediction of protein subcellular localization. Proteins.

[117] Gasteiger E (2003). ExPASy: The proteomics server for in-depth protein knowledge and analysis. Nucleic Acids Res..

[118] Almagro Armenteros JJ (2019). SignalP 5.0 improves signal peptide predictions using deep neural networks. Nat. Biotechnol..

[119] Kanehisa M, Furumichi M, Tanabe M, Sato Y, Morishima K (2017). KEGG: New perspectives on genomes, pathways, diseases and drugs. Nucleic Acids Res..

