# Supplementary material for: Mining for novel cyclomaltodextrin glucanotransferases unravels the carbohydrate metabolism pathway via cyclodextrins in Thermoanaerobacterales
Source: Sci Rep. 2022 Jan 14;12:730. doi: 10.1038/s41598-021-04569-x (PMC8760340; doi:10.1038/s41598-021-04569-x)
Supplement: Supplementary file 1 — Supplementary Figures. [file 41598_2021_4569_MOESM1_ESM.docx]

**Supplemental information to:**

**Mining for novel cyclomaltodextrin glucanotransferases unravels the carbohydrate metabolism pathway via cyclodextrins in Thermoanaerobacterales**

Sara Centeno-Leija^a*^, Laura Espinosa-Barrera^b^, Beatriz Velázquez-Cruz^b^, Yair Cárdenas-Conejo^a^, Raúl Virgen-Ortíz^b^, Georgina Valencia-Cruz^c^, Roberto A. Saenz^d^, Yerli Marín-Tovar^e^, Saúl Gómez-Manzo^f^, Beatriz Hernández-Ochoa^g^, Luz María Rocha-Ramirez^h^, Rocío Zataraín-Palacios^i^, Juan A. Osuna-Castro^j^, Agustín López-Munguía^k^, and Hugo Serrano-Posada^a*^

^a^Consejo Nacional de Ciencia y Tecnología, Laboratorio de Biología Sintética, Estructural y Molecular, Laboratorio de Agrobiotecnología, Tecnoparque CLQ, Universidad de Colima, Carretera Los Limones-Loma de Juárez, 28627 Colima, Colima, Mexico; ^b^Laboratorio de Biología Sintética, Estructural y Molecular, Laboratorio de Agrobiotecnología, Tecnoparque CLQ, Universidad de Colima, Carretera Los Limones-Loma de Juárez, 28627 Colima, Colima, Mexico; ^c^Centro Universitario de Investigaciones Biomédicas, Universidad de Colima, Avenida 25 de julio 965, Colonia Villa de San Sebastián, 28045 Colima, Colima, Mexico; ^d^Facultad de Ciencias, Universidad de Colima, Bernal Díaz del Castillo 340, 28045 Colima, Colima, Mexico; ^e^Laboratorio de Bioquímica Estructural, Departamento de Medicina Molecular y Bioprocesos, Instituto de Biotecnología, Universidad Nacional Autónoma de México, Avenida Universidad 2001, Colonia Chamilpa, 62210 Cuernavaca, Mexico; ^f^Laboratorio de Bioquímica Genética, Instituto Nacional de Pediatría, Secretaría de Salud, 04530 Ciudad de México, Mexico; ^g^Laboratorio de Inmunoquímica y Biología Celular, Hospital Infantil de México Federico Gómez, Secretaría de Salud, 06720 Ciudad de México, Mexico; ^h^Unidad de Investigación en Enfermedades Infecciosas, Hospital Infantil de México Federico Gómez, Dr. Márquez No. 162, Colonia Doctores, Delegación Cuauhtémoc 06720, Mexico; ^i^Escuela de Medicina General, Universidad José Martí, Bosques del Decán 351, 28089 Colima, Colima, México; ^j^Facultad de Ciencias Biológicas y Agropecuarias, Universidad de Colima, Autopista Colima-Manzanillo, 28100 Tecomán, Colima, Mexico; ^k^Instituto de Biotecnología, Universidad Nacional Autónoma de México, Avenida Universidad 2001, Colonia Chamilpa, 62210 Cuernavaca, Morelos, Mexico.

^*^Corresponding author:

S. Centeno-Leija, Consejo Nacional de Ciencia y Tecnología, Laboratorio de Biología Sintética, Estructural y Molecular, Laboratorio de Agrobiotecnología, Tecnoparque CLQ, Universidad de Colima, Carretera Los Limones-Loma de Juárez, 28627 Colima, Colima, Mexico; Tel. +52 3123161125 ext. 40008; Fax +52 3123161125 ext. 40002; email: [scenteno0@ucol.mx](mailto:scenteno0@ucol.mx)

H. Serrano-Posada, Consejo Nacional de Ciencia y Tecnología, Laboratorio de Biología Sintética, Estructural y Molecular, Laboratorio de Agrobiotecnología, Tecnoparque CLQ, Universidad de Colima, Carretera Los Limones-Loma de Juárez, 28627 Colima, Colima, Mexico; Tel. +52 3123161125 ext. 40008; Fax +52 3123161125 ext. 40002; email: [hserrano0@ucol.mx](mailto:hserrano0@ucol.mx)


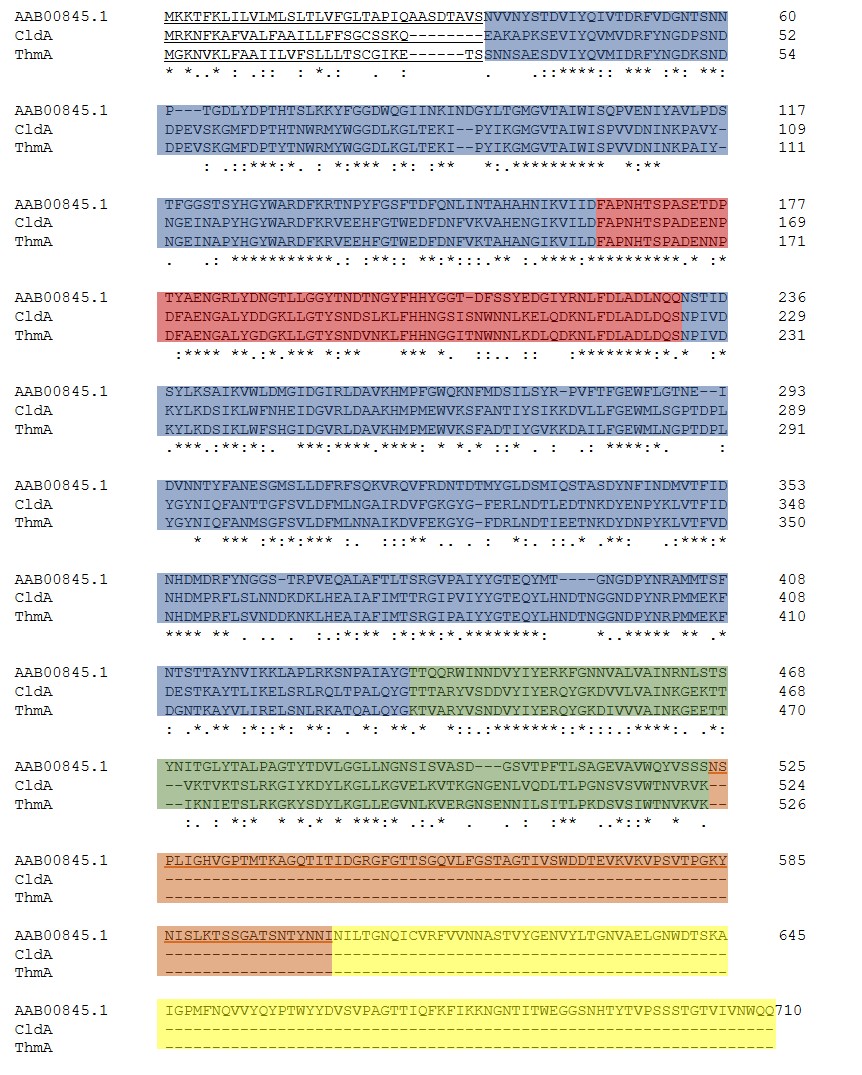
**FIG S1 Amino acid sequence alignment of CGTases from GH13_2.** The conventional five-domain ABCDE_CBM20_ CGTase from *T. thermosulfurigenes* (NCBI ID: AAB00845.1) and both three-domain ABC CGTases CldA and ThmA are shown. Note the catalytic AB domains (blue and red, respectively), the starch-binding C domain (green), D domain (orange), and E_CBM20_ domain (yellow). The signal peptide sequences are underlined.


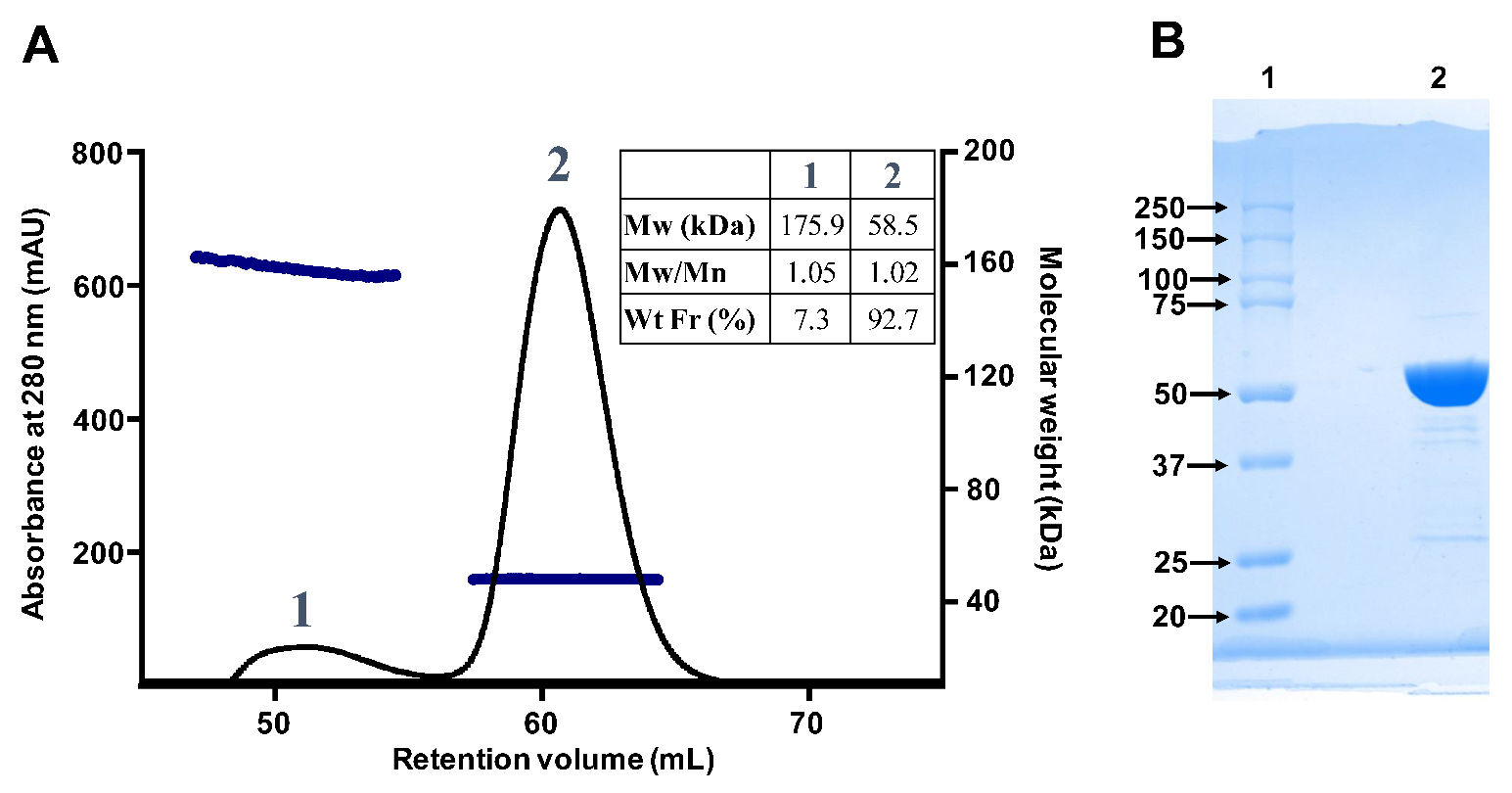


**FIG S2 Purification of recombinant CldA.** (A) SEC-DLS coupled experiment of CldA. Right inset: molecular weight (Mw), polydispersity index (Mw/Mn), and weight fraction (Wt Fr) of chromatographic peaks 1 and 2. Note that the monomer of CldA (58.5 kDa, peak 2) and the CldA aggregates (175.9 kDa, peak 1) correspond to 92.7% and 7.3% of the total injected protein, respectively. (B) Coomassie Blue-stained SDS-PAGE gel (12%) of the purified CldA (peak 2) after a heat treatment procedure, Ni^2+^-affinity chromatography, and SEC-DLS coupled experiment. Lane 1, molecular-weight markers (Bio-Rad, labeled in kDa). Lane 2, purified CldA with optimal monodispersity (Mw/Mn = 1.02, peak 2).


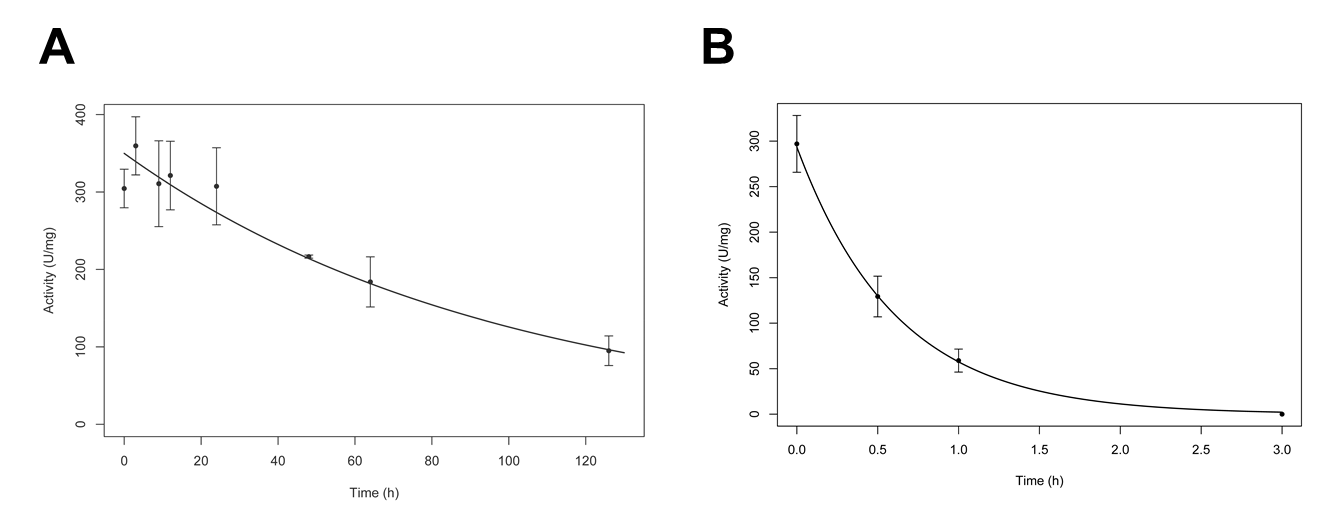


**FIG S3 CldA activity decay over time**. **(A)** 70 °C. **(B)** 80 °C.


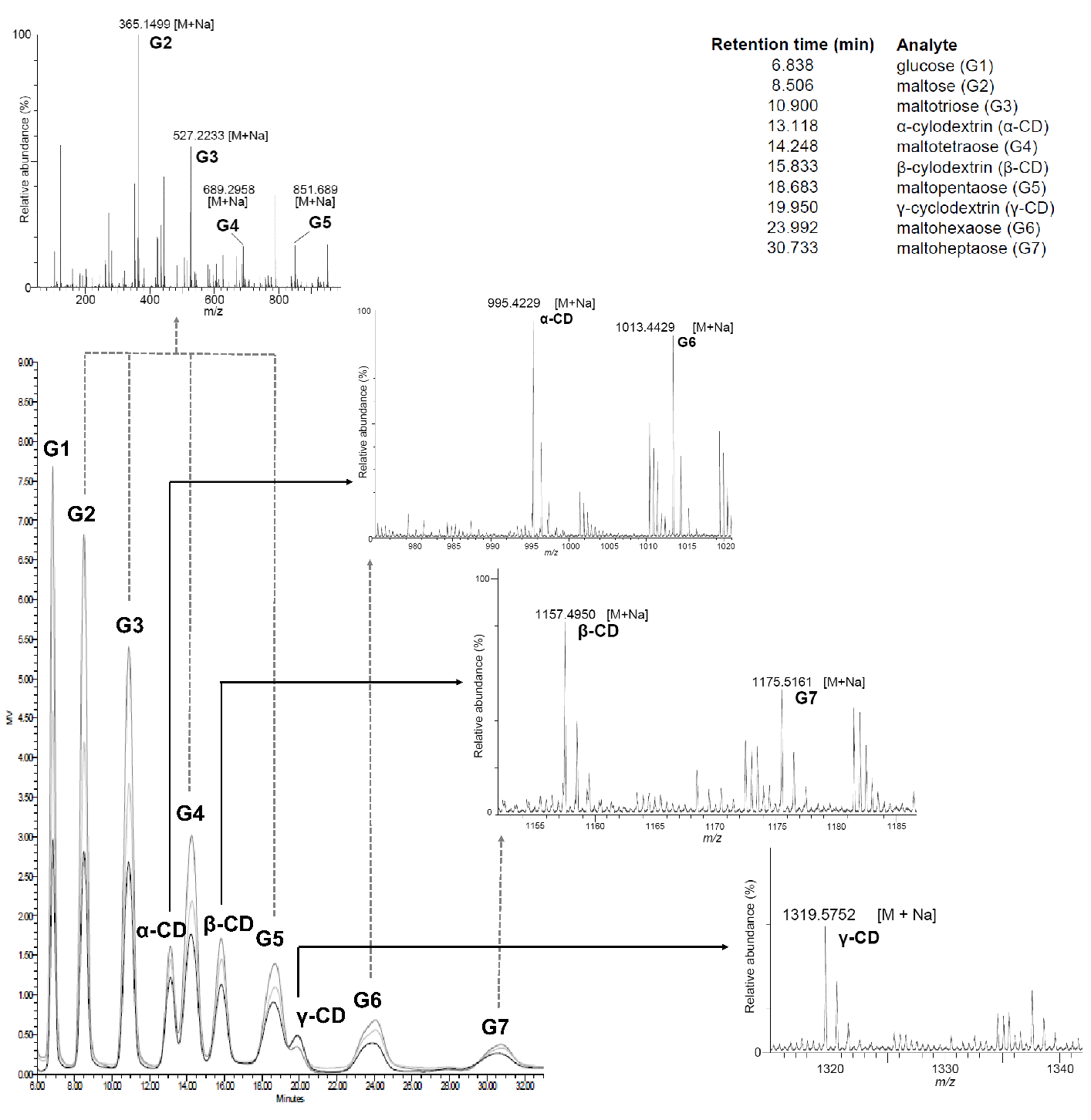


**FIG S4. Profile of end products.** HPLC and TOF MS ES+ analysis of products from 5% (*w/v*) soluble starch by the action of CldA at 75 °C and pH 4.0 for 0.5 h (black line), 1 h (light gray line), and 2 h (bold gray line). Peaks: glucose (G1), maltose (G2), maltotriose (G3), maltotetraose (G4), maltopentaose (G5), maltohexaose (G6), maltoheptaose (G7), α-cyclodextrin (α-CD), β-cyclodextrin (β-CD), and γ-cyclodextrin (γ-CD). Insets: TOF MS
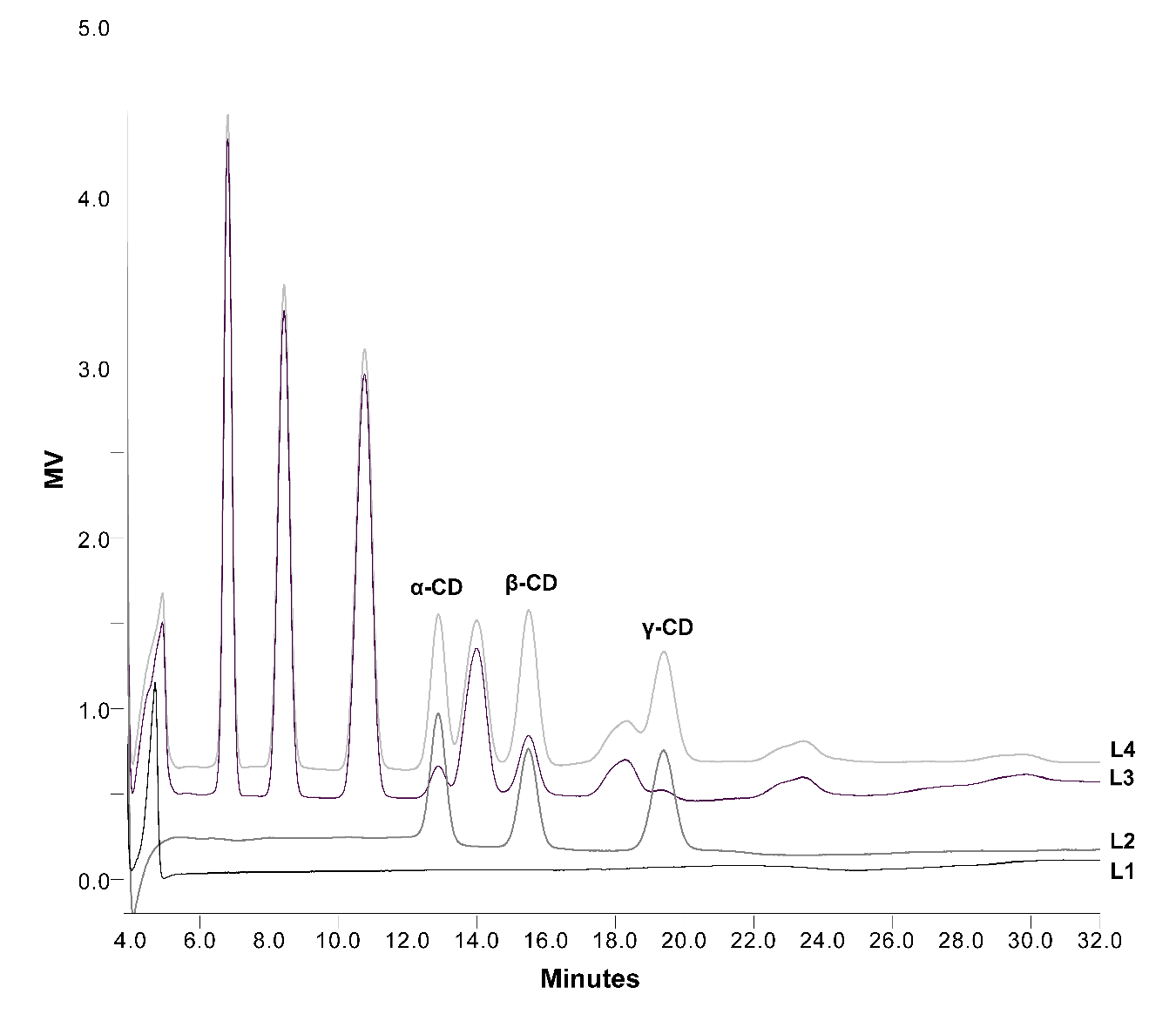
ES+ analysis of products was obtained from the mixture at 2 h (bold gray line).

**FIG S5**. **Detection of CDs from soluble starch by the action of CldA.** HPLC analysis of products from 5% (*w/v*) soluble starch at 75 °C and pH 4.0. L1: soluble starch incubated without enzyme for 2 h of reaction. L2: mixture of α-, β-, and γ-CDs standards (0.2 mg mL^-1^). L3: profile of end products from soluble starch by the action of CldA for 15 min of reaction. L4: co-elution of end products from soluble starch by the action of CldA for 15 min of reaction with a mixture of α-, β-, and γ-CDs standards (L2 + L3).


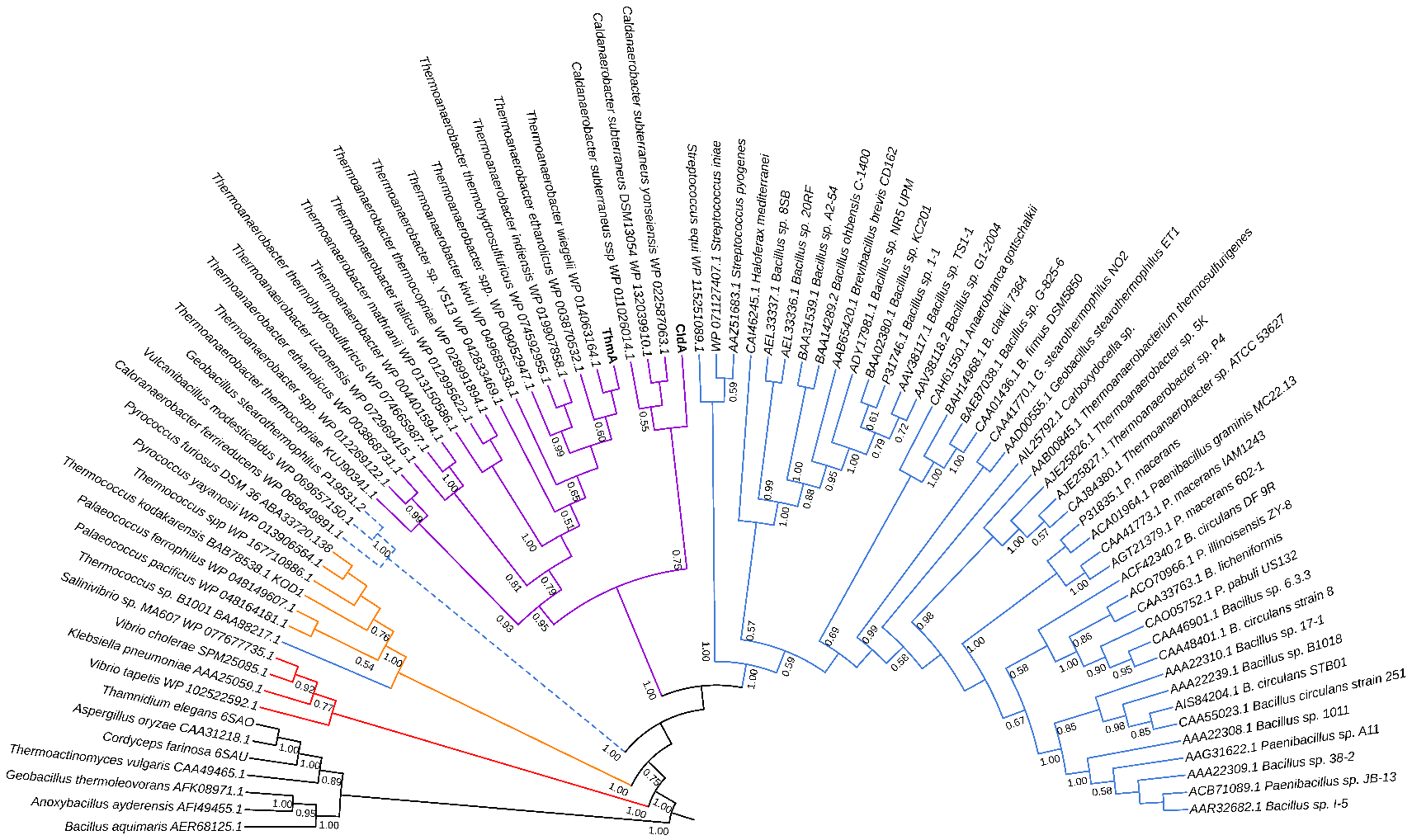


**FIG S6 Phylogenetic analysis of novel three-domain ABC CGTases**. Evolutionary relationships were determined by the maximum likelihood method based on the WAG + G model using the amino acid sequences corresponding to the ABC core structure of all 78 CGTases, including the 48 characterized CGTases from GH13_2 recognized in the CAZY database, 19 three-domain ABC (CldA/ThmA)-like CGTases, and 11 putative CGTases to improve the fit of some clades. The sequences of 7 α-amylases from GH13 were used as an outgroup. The conventional five-domain ABCDE_CBM20_ CGTases (blue), five-domain ABCDE_arch_ CGTases (orange), four-domain ABCE_CBM20_ CGTases (red), and the novel group of 19 three-domain ABC CGTases, (CldA/ThmA)-like enzymes from thermophilic *C. subterraneus* ssp. and *Thermoanaerobacter* sp. (magenta) were observed in four different clades. The ABCDE_CBM20_ maltogenic starch-acting enzymes (blue dashed line) and α-amylases (black branch) from GH13_2 and GH13, respectively, are also shown in two different clades. Bootstrap values (1,000 iterations) are indicated for each node. Only bootstrap values above 50% were shown. The tree was drawn using iTOL v4 [(itol.embl.de](https://itol.embl.de/)).
