# Supplementary material for: Mining for novel cyclomaltodextrin glucanotransferases unravels the carbohydrate metabolism pathway via cyclodextrins in Thermoanaerobacterales
Source: Sci Rep. 2022 Jan 14;12:730. doi: 10.1038/s41598-021-04569-x (PMC8760340; doi:10.1038/s41598-021-04569-x)
Supplement: Supplementary file 2 — Supplementary Table S1. [file 41598_2021_4569_MOESM2_ESM.docx]

**Supplementary Table S1.** Metagenomes of microbial communities from diverse thermophilic environments.

| **Type of thermophilic ecological niche** | **Genome Name / Sample Name** | **Origin** | **IMG Genome ID** | **Genome Size**  **(total bases)** | **Gene Count (total genes)** | **CGTases predicted by CDD/SPARCLE** | **Gene ID** | **Domain distribution** | **Identity**  **(%)** | **Possibly related microorganism** |
| --- | --- | --- | --- | --- | --- | --- | --- | --- | --- | --- |
| **Hot springs** |  |  |  |  |  |  |  |  |  |  |
| **1** | Hot spring microbial communities from Jinze hot spring, China to study Microbial Dark Matter (Phase II) - JNZ 110809A | China | 3300007999 | 218561379 | 454360 | 0 |  |  |  |  |
| **2** | Hot spring microbial communities from Gongxiaoshe hot spring, China to study Microbial Dark Matter (Phase II) - GXS 110809B | China | 3300009585 | 156747795 | 308249 | 0 |  |  |  |  |
| **3** | Hot spring microbial communities from Jinze hot spring, China to study Microbial Dark Matter (Phase II) - JNZ 110809A (SPAdes) | China | 3300025090 | 159721048 | 243959 | 0 |  |  |  |  |
| **4** | Hot spring microbial communities from South Africa to study Microbial Dark Matter (Phase II) - Sagole hot spring metaG (SPAdes) | South Africa | 3300025116 | 354346899 | 784839 | **1** | Ga0209207_10041881 | ABCE_CBM20_ | 71.2% with WP_086836998.1 | *Penibacillus* sp. |
| **5** | Hot spring sediment bacterial and archeal communities from British Columbia, Canada, to study Microbial Dark Matter (Phase II) - Dewar Creek DC2 2012 metaG (SPAdes) | Canada | 3300025775 | 179603298 | 232836 | **1** | Ga0209276_10015571 | ABCDE_CBM20_ | 99.7% with HEI01377.1 | Chloroflexi |
| **6** | Hot spring sediment bacterial and archeal communities from British Columbia, Canada, to study Microbial Dark Matter (Phase II) - Dewar Creek DC8 2012 metaG (SPAdes) | Canada | 3300025094 | 215279623 | 286210 | **1** | Ga0209478_10048853 | ABCDE_CBM20_ | 100% with HDY05196.1 | Chloroflexi |
| **7** | Hot spring sediment bacterial and archeal communities from British Columbia, Canada, to study Microbial Dark Matter (Phase II) - Larsen N4 metaG (SPAdes) | Canada | 3300025310 | 1283102380 | 2246436 | 0 |  |  |  |  |
| **8** | Hot spring microbial communities from Sandy's Spring West, USA to study Microbial Dark Matter (Phase II) - SSWsed_130316 | USA | 3300009591 | 233530326 | 618870 | 0 |  |  |  |  |
| **9** | Hot spring water microbial communities from Wilbur Hot Springs, California, USA - Wilbur Geyser funnel - 6/17/14 | USA | 3300003649 | 27908441 | 52038 | 0 |  |  |  |  |
| **10** | Hot spring sediment microbial communities from Geyser Creek Basin, Yellowstone National Park, WY, United States - GCR.EP_S | USA | 3300033490 | 206359644 | 399680 | 0 |  |  |  |  |
| **11** | Hot spring water microbial communities from Geyser Creek Basin, Yellowstone National Park, WY, United States - GCR.EP_P | USA | 3300033431 | 213599432 | 526244 | 0 |  |  |  |  |
| **12** | Hot spring water microbial communities from Geyser Creek Basin, Yellowstone National Park, WY, United States - GCR.EP_P | USA | 3300033431 | 213599432 | 526244 | 0 |  |  |  |  |
| **13** | Hot spring sediment microbial communities from Geyser Creek Basin, Yellowstone National Park, WY, United States - GCR.EP_S | USA | 3300033490 | 206359644 | 399680 | 0 |  |  |  |  |
| **14** | Hot spring sediment bacterial and archeal communities from California, USA to study Microbial Dark Matter (Phase II) - Elbow Spring sediment metaG | USA | 3300010258 | 28851082 | 69015 | 0 |  |  |  |  |
| **15** | Hot spring microbial communities from Joseph's Coat, Yellowstone National Park, USA - JC2_E | USA | 3300001684 | 131966625 | 297298 | 0 |  |  |  |  |
| **16** | Hot spring microbial communities from Joseph's Coat, Yellowstone National Park, USA - JC2_E (SPADES assembly) | USA | 3300005859 | 153229826 | 288266 | 0 |  |  |  |  |
| **17** | Thermal spring microbial communities from Yellowstone National Park, Wyoming, USA - Perpetual Spouter A (PS_A) MetaG (SPADES assembly) | USA | 3300005851 | 73012051 | 105945 | 0 |  |  |  |  |
| **18** | Thermal spring microbial communities from Yellowstone National Park, Wyoming, USA - Perpetual Spouter A (PS_A) MetaG | USA | 3300003604 | 62639124 | 104828 | 0 |  |  |  |  |
| **19** | Thermal spring microbial communities from Yellowstone National Park, Wyoming, USA - Perpetual Spouter A (PS_A) MetaG (SPAdes) | USA | 3300025345 | 69503882 | 101870 | 0 |  |  |  |  |
| **20** | Thermal spring microbial communities from Yellowstone National Park, Wyoming, USA - Perpetual Spouter B (PS_B) MetaG (SPADES assembly) | USA | 3300005852 | 85136290 | 117344 | 0 |  |  |  |  |
| **21** | Thermal spring microbial communities from Yellowstone National Park, Wyoming, USA - Perpetual Spouter B (PS_B) MetaG | USA | 3300003598 | 73220362 | 118677 | 0 |  |  |  |  |
| **22** | Thermal spring microbial communities from Yellowstone National Park, Wyoming, USA - Perpetual Spouter B (PS_B) MetaG (SPAdes) | USA | 3300025349 | 80754650 | 111707 | 0 |  |  |  |  |
| **23** | Hot spring sediment microbial communities from Zodletone spring, Oklahoma to study Microbial Dark Matter (Phase II) - Zodletone Spring source 2m metaG | USA | 3300010317 | 405129816 | 958808 | 0 |  |  |  |  |
| **24** | Hot spring sediment microbial communities from Great Boiling Spring, Nevada - Cellulolytic enrichment Sediment 77C | USA | 3300005298 | 150187695 | 231170 | 0 |  |  |  |  |
| **25** | Hot spring phototrophic mat microbial communities from Octopus Spring, Yellowstone National Park, Wyoming, United States - 20060914_OS-M3 | USA | 3300031980 | 267257994 | 617247 | 0 |  |  |  |  |
| **26** | Hot spring microbial communities from Little Hot Creek, USA to study Microbial Dark Matter (Phase II) - LHC4sed_replicate (SPAdes) | USA | 3300025068 | 145292550 | 280398 | 0 |  |  |  |  |
| **27** | Hot spring microbial communities from Yellowstone National Park, Wyoming, USA - YNP9 Dragon Spring, Norris Geyser Basin | USA | 2022920010 | 13542958 | 20567 | 0 |  |  |  |  |
| **28** | Hot spring microbial communities from Yellowstone National Park, Wyoming, USA - YNP7 Chocolate Pots | USA | 2022920013 | 27959912 | 41922 | 0 |  |  |  |  |
| **29** | Hot spring phototrophic mat microbial communities from Mushroom Spring, Yellowstone National Park, Wyoming, United States - 20060912_MS13 | USA | 3300031875 | 369211308 | 763354 | 0 |  |  |  |  |
| **30** | Hot spring phototrophic mat microbial communities from Mushroom Spring, Yellowstone National Park, Wyoming, United States - 20050930_P4 | USA | 3300031245 | 368404207 | 717367 | 0 |  |  |  |  |
| **31** | Hot spring microbial communities from Elkhorn Slough, Monterey Bay, USA - MD2A | USA | 3300000919 | 173570670 | 399161 | 0 |  |  |  |  |
| **32** | Hot spring water microbial communities from Norris-Mammoth Corridor, Yellowstone National Park, WY, United States - NMC.RSW_P | USA | 3300033894 | 147463480 | 268501 | 0 |  |  |  |  |
| **33** | Hot spring microbial mat communities from California, USA to study Microbial Dark Matter (Phase II) - Cone Pool mat layer E metaG (SPAdes) | USA | 3300025800 | 246538505 | 527772 | 0 |  |  |  |  |
| **34** | Hot spring thermophilic microbial communities from Obsidian Pool, Yellowstone National Park, USA - site 3 B9 (version 2) | USA | 3300005209 | 7200399 | 7802 | **1** | Ga0063234_1012181 | ABC | 98.8% with WP_022587063.1 | *C. subterraneus* ssp. |
| **Geysers**  **Geothermal fumarole** | |  |  |  |  |  |  |  |  |  |
| **35** | Geothermal fumarole subsurface microbial communities from Mt. Erebus, Antarctica - 1A2E | Antarctica | 3300002480 | 2494345 | 4180 | 0 |  |  |  |  |
| **36** | Geothermal fumarole subsurface microbial communities from Mt. Erebus, Antarctica - 1A2A (SPAdes) | Antarctica | 3300026949 | 6723797 | 11323 | 0 |  |  |  |  |
| **37** | Geothermal fumarole subsurface microbial communities from Mt. Erebus, Antarctica - 1A2B (SPAdes) | Antarctica | 3300026950 | 13512936 | 22054 | 0 |  |  |  |  |
| **38** | Geothermal fumarole subsurface microbial communities from Mt. Erebus, Antarctica - 1A2D | Antarctica | 3300002474 | 2544753 | 4410 | 0 |  |  |  |  |
| **39** | Geothermal fumarole subsurface microbial communities from Mt. Erebus, Antarctica - 1A2C | Antarctica | 3300002479 | 4099718 | 6684 | 0 |  |  |  |  |
| **40** | Geothermal fumarole subsurface microbial communities from Mt. Erebus, Antarctica - 1A2B | Antarctica | 3300002473 | 11221925 | 18183 | 0 |  |  |  |  |
| **41** | Geothermal fumarole subsurface microbial communities from Mt. Erebus, Antarctica - 1A2D (SPAdes) | Antarctica | 3300026654 | 5572302 | 15989 | 0 |  |  |  |  |
| **42** | Geothermal fumarole subsurface microbial communities from Mt. Erebus, Antarctica - 1A2E (SPAdes) | Antarctica | 3300027408 | 5524172 | 14098 | 0 |  |  |  |  |
| **43** | Geothermal fumarole subsurface microbial communities from Mt. Erebus, Antarctica - 1A2A | Antarctica | 3300002472 | 5406195 | 9286 | 0 |  |  |  |  |
| **44** | Groundwater microbial communities from aquifer - Crystal Geyser CG22_combo_CG10-13_8/21/14_all (SPAdes) | USA | 3300025850 | 733976360 | 1256169 | 0 |  |  |  |  |
| **45** | Groundwater microbial communities from Crystal Geyser aquifers in Utah, USA - Crystal Geyser metaG 2015-23 (SPAdes) | USA | 3300025020 | 434125281 | 756987 | 0 |  |  |  |  |
| **46** | Hypoxic/sulfidic aquatic microbial communities from Monarch Geyser, Yellowstone National Park, USA - MG (SPADES assembly) | USA | 3300005959 | 76306907 | 147621 | 0 |  |  |  |  |
| **47** | Ferric oxide microbial mat and aquatic microbial communities from Rainbow Spring, Yellowstone National Park, USA - RS3B (SPADES assembly) | USA | 3300005861 | 391079800 | 623474 | 0 |  |  |  |  |
| **Hydrothermal Vents** |  |  |  |  |  |  |  |  |  |  |
| **48** | Hydrothermal vent microbial communities from Mid Atlantic Ridge, Atlantic Ocean - 355-202 | mid-oceanic Atlantic ridge | 3300026510 | 358573039 | 562563 | 0 |  |  |  |  |
| **49** | Hydrothermal vent microbial communities from Mid Atlantic Ridge, Atlantic Ocean - 356-308 | mid-oceanic Atlantic ridge | 3300026534 | 665474504 | 1170547 | 0 |  |  |  |  |
| **50** | Hydrothermal vent microbial communities from Mid Atlantic Ridge, Atlantic Ocean - 356-284 | mid-oceanic Atlantic ridge | 3300028398 | 596509297 | 1061789 | 0 |  |  |  |  |
| **51** | Hydrothermal vent microbial communities from Mid Atlantic Ridge, Atlantic Ocean - 354-166 | mid-oceanic Atlantic ridge | 3300026519 | 577576218 | 929962 | **1** | Ga0256839_10420151; Ga0256839_10535381 | ABCDE_CBM20_ | 86% with RMF74333.1; | α-proteobacteria |
| **52** | Hydrothermal vent microbial communities from East Pacific Rise, Pacific Ocean - CV88 | mid-oceanic Pacific ridge | 3300026521 | 395783452 | 750764 | 0 |  |  |  |  |
| **53** | Hydrothermal vent microbial communities from East Pacific Rise, Pacific Ocean - CV67 | mid-oceanic Pacific ridge | 3300028490 | 611481128 | 1337530 | 0 |  |  |  |  |
| **54** | Hydrothermal vent microbial communities from East Pacific Rise, Pacific Ocean - Teddybear | mid-oceanic Pacific ridge | 3300028031 | 604341212 | 1269686 | 0 |  |  |  |  |
| **55** | Hydrothermal vent microbial communities from East Pacific Rise, Pacific Ocean - PIR-30 | mid-oceanic Pacific ridge | 3300026531 | 565819585 | 1094647 | 0 |  |  |  |  |
| **56** | Hydrothermal vent microbial communities from East Pacific Rise, Pacific Ocean - CV84 | mid-oceanic Pacific ridge | 3300026544 | 741067616 | 1466353 | 0 |  |  |  |  |
| **57** | Hydrothermal vent microbial communities from East Pacific Rise, Pacific Ocean - CV79 | mid-oceanic Pacific ridge | 3300026533 | 612734278 | 1223151 | 0 |  |  |  |  |
| **58** | Hydrothermal vent microbial communities from East Pacific Rise, Pacific Ocean - CV74 | mid-oceanic Pacific ridge | 3300037333 | 257383960 | 407587 | 0 |  |  |  |  |
| **59** | Pompeii worm associated microbial communities from hydrothermal vent at the East Pacific Rise, Pacific Ocean - Alvinella | mid-oceanic Pacific ridge | 3300028534 | 584750284 | 1130233 | 0 |  |  |  |  |
| **60** | Mussel associated microbial communities from hydrothermal vent at the East Pacific Rise, Pacific Ocean - Mussels | mid-oceanic Pacific ridge | 3300028042 | 626608301 | 1191976 | 0 |  |  |  |  |
| **61** | Tube worm associated microbial communities from hydrothermal vent at the East Pacific Rise, Pacific Ocean - Tevnia | mid-oceanic Pacific ridge | 3300028026 | 440506227 | 703030 | 0 |  |  |  |  |
| **62** | Tube worm associated microbial communities from hydrothermal vent at the East Pacific Rise, Pacific Ocean - Riftia | mid-oceanic Pacific ridge | 3300028029 | 592228120 | 1094814 | 0 |  |  |  |  |
| **63** | Hydrothermal vent microbial communities from East Pacific Rise, Pacific Ocean - 4281-140 | mid-oceanic Pacific ridge | 3300028417 | 406215226 | 718778 | 0 |  |  |  |  |
| **64** | Hydrothermal vent microbial communities from Lau Basin, Pacific Ocean - 128-326 | Australian-Pacific | 3300028399 | 512596221 | 922795 | **1** | Ga0256830_10213052 | ABCDE_CBM20_ | 78.6% with MBE7531072.1 | Ardenticatenaceae |
| **65** | Hydrothermal vent microbial communities from Lau Basin, Pacific Ocean - 134-614 | Australian-Pacific | 3300028400 | 762786935 | 1402025 | 0 |  |  |  |  |
| **66** | Hydrothermal vent microbial communities from Lau Basin, Pacific Ocean - 131-447 | Australian-Pacific | 3300026518 | 408311357 | 685938 | 0 |  |  |  |  |
| **67** | Hydrothermal vent microbial communities from Lau Basin, Pacific Ocean - 132-544 | Australian-Pacific | 3300028030 | 640257898 | 1272930 | **1** | Ga0256831_100018415 | ABCDE_arch_ | 76% with WP_088854852.1 | *Thermococcus* sp. |
| **68** | Hydrothermal chimney microbial communities from Main Endeavour vent field at the Juan de Fuca Ridge, Pacific Ocean - Hulk | Juan de Fuca Ridge-Pacific | 3300028908 | 555852493 | 943778 | **2** | Ga0256914_100550510;  Ga0256914_10008906 | ABCDE_CBM20;_  ABCDE_arch_ | 75% with BAA88217.1; 75% with WP_048164181.1 | *Thermococcus* sp.  *Paleococcus* sp. |
| **69** | Marine microbial communities from the Lost City Hydrothermal Field | USA | 3300003136 | 24827591 | 45285 | 0 |  |  |  |  |
| **70** | Marine hydrothermal vent microbial communities from Guaymas Basin, Gulf of California to study Microbial Dark Matter (Phase II) - Marker 14 Mat core 4571-4 3-6 cm metaG (SPAdes) | Mexico | 3300025163 | 612561596 | 1305276 | 0 |  |  |  |  |
| **71** | Marine hydrothermal vent microbial communities from Guaymas Basin, Gulf of California to study Microbial Dark Matter (Phase II) - Marker 14 Mat core 4571-4 33-36 cm metaG | Mexico | 3300010264 | 13337508 | 30532 | 0 |  |  |  |  |
| **72** | Marine hydrothermal vent microbial communities from Guaymas Basin, Gulf of California to study Microbial Dark Matter (Phase II) - Marker 14 Mat core 4569-2 3-6 cm metaG (SPAdes) | Mexico | 3300025156 | 944352716 | 2329548 | 0 |  |  |  |  |
| **73** | Marine hydrothermal vent microbial communities from Guaymas Basin, Gulf of California to study Microbial Dark Matter (Phase II) - Marker 14 Mat core 4571-4 33-36 cm metaG (SPAdes) | Mexico | 3300025758 | 14148566 | 34631 | 0 |  |  |  |  |
| **74** | Marine hydrothermal vent microbial communities from Guaymas Basin, Gulf of California to study Microbial Dark Matter (Phase II) - Marker 14 Mat core 4571-4 3-6 cm metaG | Mexico | 3300010332 | 1080322849 | 2490314 | 0 |  |  |  |  |
| **75** | Marine hydrothermal vent microbial communities from Guaymas Basin, Gulf of California to study Microbial Dark Matter (Phase II) - Marker 14 Mat core 4569-2 3-6 cm metaG | Mexico | 3300010330 | 1159571654 | 2483439 | 0 |  |  |  |  |
| **76** | Hydrothermal vent microbial mat bacterial communities from Southern Trench, Guaymas Basin, Mexico - 4872-13-1-2_MG | Mexico | 3300021496 | 136289761 | 306073 | 0 |  |  |  |  |
| **77** | Hydrothermal vent microbial mat bacterial communities from Southern Trench, Guaymas Basin, Mexico - 4872-13-5-6_MG | Mexico | 3300021500 | 159679707 | 329643 | 0 |  |  |  |  |
| **78** | Hydrothermal vent microbial mat bacterial communities from Southern Trench, Guaymas Basin, Mexico - 4872-13-13-14_MG | Mexico | 3300021589 | 44940317 | 121160 | 0 |  |  |  |  |
| **79** | Hydrothermal vent microbial mat bacterial communities from Southern Trench, Guaymas Basin, Mexico - 4872-13-9-10_MG | Mexico | 3300021587 | 53786313 | 138457 | 0 |  |  |  |  |
| **80** | Hydrothermal vent sediment bacterial communities from Southern Trench, Guaymas Basin, Mexico - 4872-04-11-12_MG | Mexico | 3300021591 | 129332765 | 319226 | 0 |  |  |  |  |
| **81** | Hydrothermal vent sediment bacterial communities from Southern Trench, Guaymas Basin, Mexico - 4870-07-10-11_MG | Mexico | 3300022188 | 369060282 | 766490 | 0 |  |  |  |  |
| **82** | Hydrothermal vent sediment bacterial communities from Southern Trench, Guaymas Basin, Mexico - 4870-07-0-1_MG | Mexico | 3300021512 | 346764346 | 732227 | 0 |  |  |  |  |
| **83** | Hydrothermal vent sediment bacterial communities from Southern Trench, Guaymas Basin, Mexico - 4870-11-1-2_MG | Mexico | 3300021507 | 264224592 | 551683 | 0 |  |  |  |  |
| **84** | Hydrothermal vent sediment bacterial communities from Southern Trench, Guaymas Basin, Mexico - 4872-04-13-14_MG | Mexico | 3300021567 | 20745460 | 62316 | 0 |  |  |  |  |
| **85** | Hydrothermal vent sediment bacterial communities from Southern Trench, Guaymas Basin, Mexico - 4870-07-2-3_MG | Mexico | 3300021488 | 116921597 | 241508 | 0 |  |  |  |  |
| **86** | Hydrothermal vent sediment bacterial communities from Southern Trench, Guaymas Basin, Mexico - 4872-04-4-5_MG | Mexico | 3300021483 | 99749804 | 214654 | 0 |  |  |  |  |
| **87** | Hydrothermal vent microbial mat bacterial communities from Southern Trench, Guaymas Basin, Mexico - 4869-30-0-1_MG | Mexico | 3300021514 | 378970519 | 782903 | 0 |  |  |  |  |
| **88** | Hydrothermal vent sediment bacterial communities from Southern Trench, Guaymas Basin, Mexico - 4870-07-3-4_MG | Mexico | 3300021493 | 144589974 | 293536 | 0 |  |  |  |  |
| **89** | Hydrothermal vent sediment bacterial communities from Southern Trench, Guaymas Basin, Mexico - 4872-04-0-1_MG | Mexico | 3300021505 | 158973755 | 401829 | 0 |  |  |  |  |
| **90** | Hydrothermal vent microbial mat bacterial communities from Southern Trench, Guaymas Basin, Mexico - 4872-13-2-3_MG | Mexico | 3300021504 | 241930256 | 486164 | 0 |  |  |  |  |
| **91** | Hydrothermal vent microbial communities from Guaymas Basin, Mexico - 4559-240 | Mexico | 3300026512 | 417525177 | 737412 | **3** | Ga0256842_10085191;  Ga0256842_10045645;  Ga0256842_10170851 | ABCDE_arch_ | 74.6% with WP_048164181.1; 82.8 % with WP_167889765.1; 92.6% with WP_088867030.1 | *Paleococcu*s sp.  *Thermococcus* sp.    *T. radiotolerans* |
| **92** | Hydrothermal vent microbial mat bacterial communities from Southern Trench, Guaymas Basin, Mexico - 4869-18-2-3_MG | Mexico | 3300021588 | 95272852 | 208464 | 0 |  |  |  |  |
| **93** | Hydrothermal vent sediment bacterial communities from Southern Trench, Guaymas Basin, Mexico - 4870-07-4-5_MG | Mexico | 3300021502 | 163783968 | 347638 | 0 |  |  |  |  |
| **94** | Hydrothermal vent microbial mat bacterial communities from Southern Trench, Guaymas Basin, Mexico - 4872-18-4-5_MG | Mexico | 3300021469 | 417006857 | 883977 | 0 |  |  |  |  |
| **95** | Hydrothermal vent sediment bacterial communities from Southern Trench, Guaymas Basin, Mexico - 4870-07-1--2_MG | Mexico | 3300021509 | 295641287 | 602278 | **1** | Ga0190304_10006135 | ABCDE_CBM20_ | 94.3% with BAA88217.1 | *Thermococcus* sp. |
| **96** | Hydrothermal vent sediment bacterial communities from Southern Trench, Guaymas Basin, Mexico - 4872-04-7-8_MG | Mexico | 3300021467 | 378956998 | 775933 | 0 |  |  |  |  |
| **97** | Hydrothermal vent microbial mat bacterial communities from Southern Trench, Guaymas Basin, Mexico - 4872-13-12-13_MG | Mexico | 3300021590 | 121224396 | 291648 | 0 |  |  |  |  |
| **98** | Hydrothermal vent microbial mat bacterial communities from Southern Trench, Guaymas Basin, Mexico - 4872-18-1-2_MG | Mexico | 3300021506 | 237083223 | 525249 | 0 |  |  |  |  |
| **99** | Hydrothermal vent microbial mat bacterial communities from Southern Trench, Guaymas Basin, Mexico - 4872-18-5-6_MG | Mexico | 3300021592 | 93872894 | 234219 | 0 |  |  |  |  |
| **100** | Hydrothermal vent sediment bacterial communities from Southern Trench, Guaymas Basin, Mexico - 4872-04-3-4_MG | Mexico | 3300021490 | 124187887 | 266662 | 0 |  |  |  |  |
| **101** | Hydrothermal vent sediment bacterial communities from Southern Trench, Guaymas Basin, Mexico - 4872-04-8-9_MG | Mexico | 3300021489 | 121323270 | 263884 | 0 |  |  |  |  |
| **102** | Hydrothermal vent microbial mat bacterial communities from Southern Trench, Guaymas Basin, Mexico - 4869-18-1-2_MG | Mexico | 3300021511 | 303118174 | 607177 | 0 |  |  |  |  |
| **103** | Hydrothermal vent microbial mat bacterial communities from Southern Trench, Guaymas Basin, Mexico - 4869-30-3-4_MG | Mexico | 3300021564 | 5573716 | 16695 | 0 |  |  |  |  |
| **104** | Hydrothermal vent microbial mat bacterial communities from Southern Trench, Guaymas Basin, Mexico - 4869-18-0-1_MG | Mexico | 3300021508 | 242313859 | 494266 | 0 |  |  |  |  |
| **105** | Hydrothermal vent sediment bacterial communities from Southern Trench, Guaymas Basin, Mexico - 4872-04-2-3_MG | Mexico | 3300021494 | 132448079 | 295983 | 0 |  |  |  |  |
| **106** | Hydrothermal vent microbial mat bacterial communities from Southern Trench, Guaymas Basin, Mexico - 4872-13-10-11_MG | Mexico | 3300021495 | 142764967 | 312710 | 0 |  |  |  |  |
| **107** | Hydrothermal vent microbial mat bacterial communities from Southern Trench, Guaymas Basin, Mexico - 4872-13-11-12_MG | Mexico | 3300021563 | 35541007 | 102672 | 0 |  |  |  |  |
| **108** | Hydrothermal vent microbial mat bacterial communities from Southern Trench, Guaymas Basin, Mexico - 4872-18-2-3_MG | Mexico | 3300021471 | 591576850 | 1186311 | 0 |  |  |  |  |
| **109** | Hydrothermal vent microbial mat bacterial communities from Southern Trench, Guaymas Basin, Mexico - 4869-18-3-4_MG | Mexico | 3300024423 | 431675414 | 1007877 | 0 |  |  |  |  |
| **110** | Hydrothermal vent microbial communities from Guaymas Basin, Mexico - 4561-380 | Mexico | 3300026382 | 150696482 | 273636 | 0 |  |  |  |  |
| **111** | Hydrothermal vent sediment bacterial communities from Southern Trench, Guaymas Basin, Mexico - 4870-11-0-1_MG | Mexico | 3300021513 | 332012557 | 697199 | 0 |  |  |  |  |
| **112** | Hydrothermal vent sediment bacterial communities from Southern Trench, Guaymas Basin, Mexico - 4872-04-5-6_MG | Mexico | 3300021491 | 131033271 | 278801 | 0 |  |  |  |  |
| **113** | Hydrothermal vent microbial mat bacterial communities from Southern Trench, Guaymas Basin, Mexico - 4872-18-9-10_MG | Mexico | 3300022170 | 116537782 | 266968 | 0 |  |  |  |  |
| **114** | Hydrothermal vent microbial mat bacterial communities from Southern Trench, Guaymas Basin, Mexico - 4869-30-1-2_MG | Mexico | 3300021580 | 87935217 | 241630 | 0 |  |  |  |  |
| **115** | Hydrothermal vent microbial mat bacterial communities from Southern Trench, Guaymas Basin, Mexico - 4872-13-6-7_MG | Mexico | 3300021503 | 175339565 | 362848 | 0 |  |  |  |  |
| **116** | Hydrothermal vent sediment bacterial communities from Southern Trench, Guaymas Basin, Mexico - 4872-04-6-7_MG | Mexico | 3300021482 | 104314933 | 219294 | 0 |  |  |  |  |
| **117** | Hydrothermal vent microbial mat bacterial communities from Southern Trench, Guaymas Basin, Mexico - 4872-13-3-4_MG | Mexico | 3300021484 | 106167820 | 214538 | 0 |  |  |  |  |
| **118** | Hydrothermal vent microbial mat bacterial communities from Southern Trench, Guaymas Basin, Mexico - 4872-18-10-11_MG | Mexico | 3300022470 | 345302164 | 693567 | 0 |  |  |  |  |
| **119** | Hydrothermal vent microbial mat bacterial communities from Southern Trench, Guaymas Basin, Mexico - 4872-18-3-4_MG | Mexico | 3300021468 | 516607444 | 1034703 | 0 |  |  |  |  |
| **120** | Hydrothermal vent sediment bacterial communities from Southern Trench, Guaymas Basin, Mexico - 4870-07-6-7_MG | Mexico | 3300021498 | 157464716 | 339111 | 0 |  |  |  |  |
| **121** | Hydrothermal vent microbial mat bacterial communities from Southern Trench, Guaymas Basin, Mexico - 4872-13-4-5_MG | Mexico | 3300021486 | 119658292 | 239184 | 0 |  |  |  |  |
| **122** | Hydrothermal vent sediment bacterial communities from Southern Trench, Guaymas Basin, Mexico - 4872-04-9-10_MG | Mexico | 3300021492 | 138435750 | 304837 | 0 |  |  |  |  |
| **123** | Hydrothermal vent sediment bacterial communities from Southern Trench, Guaymas Basin, Mexico - 4872-04-10-11_MG | Mexico | 3300021499 | 144358644 | 327090 | 0 |  |  |  |  |
| **124** | Hydrothermal vent microbial communities from Guaymas Basin, Mexico - 4562-384 | Mexico | 3300026484 | 263515736 | 461653 | 0 |  |  |  |  |
| **125** | Hydrothermal vent microbial communities from Guaymas Basin, Mexico - 4571-419 | Mexico | 3300026488 | 304035269 | 494359 | 0 |  |  |  |  |
| **126** | Hydrothermal vent microbial mat bacterial communities from Southern Trench, Guaymas Basin, Mexico - 4872-18-6-7_MG | Mexico | 3300021472 | 535522184 | 1088135 | 0 |  |  |  |  |
| **TOTAL GENES** |  |  |  |  | **68625500** |  |  |  |  |  |
| **TOTAL HITS** |  |  |  |  |  | **14** |  |  |  |  |
| **TOTAL CGTases**  **(not redundant)** |  |  |  |  |  | **13** |  |  |  |  |
|  |  |  |  |  |  |  |  |  |  |  |
|  |  |  |  |  |  |  |  |  |  |  |
|  |  |  |  |  |  |  |  |  |  |  |
