# Supplementary material for: Mining for novel cyclomaltodextrin glucanotransferases unravels the carbohydrate metabolism pathway via cyclodextrins in Thermoanaerobacterales
Source: Sci Rep. 2022 Jan 14;12:730. doi: 10.1038/s41598-021-04569-x (PMC8760340; doi:10.1038/s41598-021-04569-x)
Supplement: Supplementary file 3 — Supplementary Table S2. [file 41598_2021_4569_MOESM3_ESM.docx]

**Supplementary Table S2.** Metagenomes of microbial communities from Obsidian Pool hot spring at Yellowstone National Park (Wyoming, USA).

|  | **IMG Genome ID** | **Genome Size**  **(Number of total bases)*** | **Gene Count**  **(Number of total genes)*** | **Putative three-domain ABC CGTase**  **(Gene ID)** | **Scaffold ID/length** | **Identity with CldA**  **(%)** | **Identity with ThmA**  **(%)** |
| --- | --- | --- | --- | --- | --- | --- | --- |
| 1 | 2016842005 | 24421805 | 38984 | ND |  |  |  |
| 2 | 2022920021 | 18742162 | 31932 | ND |  |  |  |
| 3 | 3300001986 | 8676194 | 11634 | JGI24718J22297_10007019 (CldA) | JGI24718J22297_100070 (22336 bp) | 99.90 | 81.30 |
| 4 | 3300001987 | 12920070 | 14782 | ND |  |  |  |
| 5 | 3300002085 | 5693260 | 6797 | ND |  |  |  |
| 6 | 3300002086 | 9946993 | 11318 | ND |  |  |  |
| 7 | 3300002182 | 849468172 | 1633191 | ND |  |  |  |
| 8 | 3300002207 | 2602104 | 2832 | ND |  |  |  |
| 9 | 3300002208 | 5684792 | 10447 | ND |  |  |  |
| 10 | 3300002539 | 14752731 | 21318 | JGI24228J36427_1000945 (ThmA) | JGI24228J36427_100094 (17866 bp) | 81.50 | 100 |
| 11 | 3300002540 | 9640893 | 14159 | ND |  |  |  |
| 12 | 3300002555 | 11216427 | 14800 | ND |  |  |  |
| 13 | 3300002556 | 10644896 | 15873 | ND |  |  |  |
| 14 | 3300002966 | 1213896977 | 2118793 | ND |  |  |  |
| 15 | 3300004269 | 10392474 | 11524 | ND |  |  |  |
| 16 | 3300005209 | 7200399 | 7802 | Ga0063234_1012181 (CldA) | Ga0063234_101 (3019019 bp) | 100 | 81.50 |
| 17 | 3300005291 | 6582149 | 6769 | ND |  |  |  |
| 18 | 3300005292 | 10372310 | 11400 | ND |  |  |  |
| 19 | 3300005396 | 6036257 | 6476 | ND |  |  |  |
| 20 | 3300005573 | 1324228739 | 1898800 | ND |  |  |  |
| 21 | 3300005847 | 4497122 | 5374 | ND |  |  |  |
| 22 | 3300005848 | 6063420 | 7509 | ND |  |  |  |
| 23 | 3300005849 | 10151071 | 11299 | ND |  |  |  |
| 24 | 3300005850 | 9205925 | 13035 | ND |  |  |  |
| 25 | 3300026776 | 12854215 | 17367 | Ga0207429_101445 (ThmA) | Ga0207429_10144 (13175 bp) | 81.50 | 100 |
| 26 | 3300026777 | 14224969 | 19280 | ND |  |  |  |
| 27 | 3300026778 | 11952922 | 13225 | ND |  |  |  |
| 28 | 3300026781 | 17281314 | 20442 | Ga0207430_1023414 (ThmA) | Ga0207430_10234 (18024 bp) | 81.50 | 100 |
| 29 | 3300027863 | 1826217283 | 3013666 |  |  |  |  |

*assembled; ND, not detected,
