# Supplementary material for: Mining for novel cyclomaltodextrin glucanotransferases unravels the carbohydrate metabolism pathway via cyclodextrins in Thermoanaerobacterales
Source: Sci Rep. 2022 Jan 14;12:730. doi: 10.1038/s41598-021-04569-x (PMC8760340; doi:10.1038/s41598-021-04569-x)
Supplement: Supplementary file 4 — Supplementary Table S3. [file 41598_2021_4569_MOESM4_ESM.docx]

**Supplementary Table S3.** Three-domain ABC CGTases detected by (meta)genome mining of extremely thermophilic microorganisms.

| **Putative three-domain ABC CGTases (NCBI ID)** | **Identity (%)^a^** | **Microorganism** | **Origin^b^** | **(Meta)genome ID** |
| --- | --- | --- | --- | --- |
| **CldA** |  |  |  |  |
| Ga0063234_1012181 | 100 | Unknown^c^ | Hot spring; Yellowstone National Park, USA | IMG/M: 3300005209^e^ |
| JGI24718J22297_10007019 |  | Unknown^c^ | Hot spring; Yellowstone National Park, USA | IMG/M: 3300001986^e^ |
| **CldA-like** |  |  |  |  |
| WP_022587063.1 | 98.9 | *C. subterraneus* ssp. *yonseiensis KB-1*  *C. subterraneus strain 1523vc* | Hot spring; Java, Indonesia  Hot spring; Kamchatka, Russia | AXDC01000002.1  JABEQB010000018.1 |
| WP_132039910.1 | 98.1 | *C. subterraneus*  *DSM 13054* | NR | SLWU00000000.1 |
| WP_011026014.1 | 98.1 | *C. subterraneus* ssp. *subterraneus*  *UBA12544* | Terrestrial metagenome; Australia | DOLB00000000.1 |
|  |  | *C. subterraneus* ssp. *subterraneus* 38_43 | Oil reservoir; Alaska, USA | LGEY01000002 |
|  |  | *C. subterraneus* ssp. *tengcongensis MB4* | Hot spring; Tengcong, China | AE008691.1 |
|  |  | *Caldanaerobacter subterraneus strain Bu81* | Thermal ecosystems; Russia | JADBKI010000006 |
|  |  | *C. subterraneus* ssp. *pacificus DSM 12653* | Submarine hot vent; Okinawa, Japan | ABXP02000099.1 |
| **ThmA** |  |  |  |  |
| Ga0207430_1023414 |  | Unknown^d^ | Hot spring; Yellowstone National Park, USA | IMG/M: 3300026781^e^ |
| JGI24228J36427_1000945 | 81.5 | Unknown^d^ | Hot spring; Yellowstone National Park, USA | IMG/M: 3300002539^e^ |
| Ga0207429_101445 |  | Unknown^d^ | Hot spring; Yellowstone National Park, USA | IMG/M: 3300026776^e^ |
| **ThmA-like** |  |  |  |  |
| WP_003870532.1 | 81.5 | *T. ethanolicus* JW 200 | Hot spring; NR | CP033580 |
| WP_014063164.1 |  | *T. wiegelii* Rt8.B1 | Hot spring; New Zealand | CP002991 |
| WP_074665987.1 | 81.8 | *T. thermohydrosulfuricus* | NR | FONI01000027.1 |
| WP_004401594.1 | 81.7 | *T. siderophilus SR4* | Hydrothermal vent; Kamchatka, Russia | CM001486.1 |
| WP_072969415.1 | 82.8 | *T. uzonensis DSM 18761* | Thermal Field; Kamchatka, Russia | FQUR01000020 |
| WP_012995622.1 | 82.3 | *T. italicus Ab9* | Thermal mud; Italy | CP001936.1 |
| WP_013150586.1 | 82.6 | *T. mathranii* ssp. *mathranii str. A3* | Hot spring; Hverdagerdi-Hengil, Iceland | CP002032.1 |
| WP_028991894.1 |  | *T. thermocopriae JCM 7501* | Compost; Japan | JADV01000009 |
| WP_074592955.1 | 81.5 | *T. thermohydrosulfuricus* | NR | FNBS01000112.1 |
| WP_019907858.1 | 81.9 | *T. indiensis BSB-33* | Hydrothermal vent; NR | ARDJ01000020 |
| WP_009052947.1 |  | *Thermoanaerobacter* sp. | Deep subsurface; Colorado, USA | ACXP02000004.1 |
| WP_012269122.1 |  | *T. pseudethanolicus ATCC 33223* | NR | CP000924.1 |
|  | 83.6 | *T. brockii ssp. finnii Ako-1* | Lake Kivu sediments; Africa | CP002466.1 |
|  |  | *Thermoanaerobacter* sp. | NR | DOPY01000054 |
| WP_003868731.1 | 83.6 | *T. ethanolicus CCSD1* | Freshwater; NR | ACXY01000044.1 |
| KUJ90341.1 | 82.6 | *T. thermocopriae* | Oil reservoir; Alaska, USA | LGEO01000024 |

^a^ Sequence identity with CldA, including the N-terminal signal peptide (100% query coverage).

^b^ According to BioSample data from the GenBank database.

^c^ Possibly related to *C. subterraneus* ssp.

^d^ Possibly related to *Thermoanaerobacter* spp.

^e^ Integrated Microbial Genomes and Microbiomes (IMG/M) database from Joint Genome Institute (JGI).

NR, not reported.
