# Supplementary material for: Mining for novel cyclomaltodextrin glucanotransferases unravels the carbohydrate metabolism pathway via cyclodextrins in Thermoanaerobacterales
Source: Sci Rep. 2022 Jan 14;12:730. doi: 10.1038/s41598-021-04569-x (PMC8760340; doi:10.1038/s41598-021-04569-x)
Supplement: Supplementary file 5 — Supplementary Table S4. [file 41598_2021_4569_MOESM5_ESM.docx]

**Supplementary Table S4.** Putative proteins encoded in the *cld* and *thm* gene clusters from *C. subterraneus* ssp. and *Thermoanarobacter* spp, respectively.

|  | **Protein function description^a^** | **Function reference** | **Proteins of known crystal structure with similar function** | **Gene ID** | | **Cellular location^b^** | **Metabolic function** | **Experimental characterization reference** |
| --- | --- | --- | --- | --- | --- | --- | --- | --- |
|  |  |  |  | *C.* *subterraneus* ssp. | *Thermoanaerobacter* spp. |  |  |  |
| **1** | CldA/ThmA  cyclomaltodextrin glucanotransferase  (CGTase; EC 2.4.1.19) | this work |  | WP_022587063.1; WP_170270995.1;  WP_011026014.1; WP_132039910.1 | WP_003868731.1; WP_012269122.1  KUJ90341.1; WP_072969415.1  WP_013150586.1; WP_009052947.1  WP_028991894.1; WP_004401594.1  WP_019907858.1; WP_012995622.1  WP_014063164.1; WP_074665987.1  WP_074592955.1; WP_003870532.1  WP_049685538.1 | Extracelullar | α-glucan metabolism | this work |
| **2** | MdxE cyclo/maltodextrin-binding protein | (8) | MdxE from *A. acidocaldarius*, and *T. vulgaris*  (PDB ID: 1URD and 2ZYK, respectively) | WP_022587064.1; WP_132039912.1  WP_009611026.1 | WP_014063163.1; WP_003870531.1; WP_072969414.1; WP_049685537.1  WP_004401593.1; WP_009052946.1  KUJ90342.1; WP_003868732.1; WP_013150585.1; WP_012995621.1 | Periplasmic | Carbohydrate transport | putative |
| **3** | MdxF cyclo/maltodextrin permease subunit | (9–11) | Trehalose permease subunit  *Mycolicibacterium smegmatis*  (PDB ID: 7CAD) | WP_031313860.1; WP_170270994.1; WP_041587183.1 | WP_003868311.1; WP_006569394.1; WP_004401592.1; WP_072969413.1; WP_012995620.1; WP_049685536.1 | Membrane | Carbohydrate transport | putative |
| **4** | MdxG cyclo/maltodextrin permease subunit | (9–11) | Trehalose permease subunit  *Mycolicibacterium smegmatis*  (PDB ID: 7CAD) | WP_022587065.1; WP_170270993.1; WP_009611027.1 | WP_003868312.1; WP_014063162.1; WP_006569393.1; WP_074592954.1; WP_012995619.1; WP_072969412.1; WP_028991895.1 | Membrane | Carbohydrate transport | putative |
| **7** | Cyclomatodextrinase  (CDase; EC 3.2.1.54) | (16) | CDase from  *Thermus* sp.  (PDB ID: 1GVI) | WP_170270989.1; WP_009611031.1  WP_132039918.1 | AAA23219.1; WP_072969408.1; WP_074592679.1  WP_003870525.1; WP_014063159.1  WP_006569389.1; WP_019907857.1  WP_012269128.1; WP_004401582.1  WP_009052942.1; WP_012995615.1  WP_049685531.1 | Cytoplasmic | α-glucan metabolism | (16) |
| **8** | Glucoamylase GH15  (GA; EC 3.2.1.3) | (34) | GA  *Thermoanaerobacterium thermosaccharolyticum*  (PDB ID: 1LF6) | WP_011026002.1; WP_132040700.1; | WP_072969402.1; EIV99447.1; WP_004401571.1; WP_003871318.1; WP_074592677.1; WP_074666190.1 | Cytoplasmic | α-glucan metabolism | (34, 35) |
| **9** | alpha-glucan phosphorylase  (GP or GlgP, EC 2.4.1.1) | (43) |  | WP_022587069.1; WP_132040706.1; WP_011025994.1; WP_170271718.1 | WP_013150565.1; WP_012995582.1; WP_049685512.1; WP_028992613.1; WP_003871156.1; WP_072968246.1; WP_006569947.1; WP_003868337.1; WP_014063141.1; WP_004400034.1; WP_074592879.1 | Cytoplasmic | α-glucan metabolism | (43) |
| **10** | Phosphoglucose isomerase  (Pgi; EC 5.3.1.9)  converting glucose-6-phosphate to fructose-6-phosphate from EMP | (42) | Pgi from  *G. stearothermophilus*  (PDB ID: 1B0Z) | WP_022587068.1; WP_011025995.1; WP_170271717.1 | WP_028992612.1; WP_013150566.1; WP_049685513.1; WP_012995583.1; WP_014063142.1; WP_072968244.1; WP_009052926.1; WP_074592880.1; WP_003868336.1; WP_004400033.1; WP_006569946.1; WP_019907848.1 | Cytoplasmic | Carbohydrate central metabolism | putative |
| **11** | 6-phosphofructokinase  (PfkA; EC 2.7.1.11)  Include conserved pair Gly104/Gly124 residues related to conventional ATP-dependent EMP pathway | (29) | PfkA  *G. stearothermophilu*s (PDB ID: 1MTO) | WP_132039924.1; WP_011026004.1 | WP_042833457.1; WP_012995608.1; WP_004401579.1; WP_003868325.1; WP_049685524.1 | Cytoplasmic | Carbohydrate central metabolism | (30) |
| **12** | Pyruvate kinase  (PykF; EC 2.7.1.40)  rate-limiting final step in EMP pathway with PEP to pyruvate yielding ATP | (31, 32) | PykF  *G. stearothermophilus, and S. mutans*  (PDB ID:  2E28 and 3T05, respectively) | WP_170270985.1; WP_009611282.1; WP_132039926.1 | WP_014063154.1; WP_003870519.1; WP_003868326.1; WP_072969404.1; WP_009052938.1; WP_042833456.1; WP_049685523.1; WP_013150582.1; WP_012995607.1; WP_028991899.1 | Cytoplasmic | Carbohydrate central metabolism | putative |
| **13** | HPr (PtsH)  PTS-mediated carbohydrate transport; catabolite repressor via CcpA/cre | (20, 21) |  | WP_011026008.1; PDB 3LE5_A  TRANSCRIPTIONAL REGULATION VIA HPr/PtsK  HPrK/P: WP_022588816.1 and WP_009052761.1  CcpA: | WP_003870522.1; WP_003868320.1; WP_049685528.1; WP_012995612.1; KUJ90352.1 | Cytoplasmic | Carbohydrate metabolism | [19–21; PDB accession 3LE5_A] |
| **14** | UDP-N-acetylmuramate dehydrogenase  (MurB; EC 1.3.1.98) Formation of UDP-N-acetylmuramic peptidoglycan biosynthesis | (1, 2) | MurB from *Staphyloccocus aureus*  (PDB ID: 1HSK) | WP_022587059.1; WP_170270998.1;  WP_132039904.1; WP_011026016.1 | WP_003868727.1; WP_009052950.1;  WP_003870536.1; WP_013150587.1;  WP_072969419.1; WP_012995626.1;  WP_028991893.1; WP_049685541.1 | Cytoplasmic | Cell wall and cellular division | putative |
| **15** | PHP-histidinol phosphatase  Modulation of cell wall-associated polysaccharides biosynthesis via phosphorelay system | (3, 4) |  | WP_170270997.1; WP_022587060.1;  WP_009611014.1 | WP_012268667.1; WP_039913482.1;  WP_072969418.1; WP_012995625.1;  WP_049686257.1; WP_004401596.1;  WP_014063166.1; WP_006569396.1;  WP_003870535.1 | Cytoplasmic | Cell wall and cellular division | putative |
| **16** | RapZ regulator-like  RNA-mediated regulatory network of glucosamine biogenesis | (5) | RapZ regulator from *E. coli* (PDB ID: 5O5O) | WP_170270996.1; WP_009611021.1;  WP_022587061.1; WP_132039906.1; | WP_003870534.1; WP_003868729.1  WP_072969417.1; WP_049685540.1  WP_012995624.1; WP_013297279.1 | Cytoplasmic | Cell wall and cellular division | putative |
| **17** | RodZ-like  Peptidoglycan elongasome-associated regulator protein and cell division regulator | (14, 15) | RodZ from *B. subtilis*  (NCBI ID: CUB57083.1) | WP_170270990.1; WP_132039916.1; WP_011026011.1; WP_009611033.1 | WP_012995616.1; WP_009052943.1; WP_006569390.1; WP_012269127.1;  WP_014063160.1; WP_003870526.1; WP_004401583.1; WP_042833463.1;  WP_072969409.1; WP_049685532.1; WP_003868315.1 | Membrane | Cell wall and cellular division | putative |
| **18** | DNA-binding WhiA  transcription activator  cell division and DNA segregation | (17–19) | WhiA from *Thermotoga maritima*  (PDB ID: 3HYI) | WP_170270987.1; WP_011026009.1; WP_132039920.1 | WP_004401581.1; WP_014063157.1  WP_003870523.1; WP_019907856.1  WP_012995613.1; WP_012269129.1  WP_074592678.1; WP_009052941.1  WP_003868319.1; WP_072969407.1  WP_049685529.1 | Cytoplasmic | Cell wall and cellular division | putative |
| **19** | 2-phospho-L-lactate transferase  (EC 2.7.8.28)  biosynthesis of methanogenic redox-active cofactor coenzyme F420 | (6, 7) |  | WP_022587062.1; WP_132039908.1  WP_011026015.1 | WP_003868730.1; WP_049685539.1  WP_004401595.1; WP_014063165.1  WP_074665985.1; WP_019907859.1  WP_012995623.1; WP_072969416.1  WP_015311140.1 | Cytoplasmic/membrane | Oxidative stress | putative |
| **20** | Component E1 (R)-2-hydroxyglutaryl-CoA dehydratase (activator HgdC-like; EC 4.2.1.167) glutamate metabolism to butyrate fermentation | (12, 13) |  | WP_170270992.1; WP_132039914.1; WP_009611030.1; WP_011026013.1 | WP_028991896.1; WP_013150584.1;  WP_012995618.1; WP_072969411.1;  WP_009052945.1; WP_006569392.1; WP_003868313.1; WP_074666194.1; WP_004401585.1; WP_049685534.1 | Cytoplasmic | Oxidative stress | putative |
| **21** | Component E2 (R)-2-hydroxyglutaryl-CoA dehydratase (HgdD-like; EC 4.2.1.167) glutamate metabolism to butyrate fermentation | (12, 13) |  | WP_170270991.1; WP_022587066.1; WP_011026012.1; KKC29300.1 | WP_012268668.1; WP_003868314.1; WP_072969410.1; WP_004401584.1;  WP_009052944.1; WP_042833464.1; WP_003870527.1; WP_012995617.1; WP_049685533.1 | Cytoplasmic | Oxidative stress | putative |
| **22** | Feruloyl esterase  (EC 3.1.1.73)  Intracellular release of feruloic and cinnamic acids from esterified oligosaccharides | (38) | Cinnamoyl esterase  *Lactobacillus johnsonii*  (PDB ID: 3PF8) | WP_011025998.1; WP_009611158.1; WP_170271822.1 | WP_014063144.1; WP_006569944.1; WP_003871325.1; WP_004401544.1;  WP_003868333.1; WP_013150560.1; WP_072968262.1; WP_009052868.1; WP_012995574.1; | Cytoplasmic | carbohydrate and aromatic metabolism | (39) |
| **23** | 4-hydroxybenzoyl-CoA thioesterase  Long- and short-chain acyl-CoA thioesters hydrolysis/ benzoate metabolism | (33) | 4HB-CoA  *Staphylococcus aureus*  (PDB ID: 6FDG) | WP_170270984.1; WP_011026003.1 | WP_012995606.1; WP_003868327.1;  WP_004401572.1; WP_049685522.1; WP_072969403.1; WP_012269131.1 | Cytoplasmic | Fatty acid metabolism | putative |
| **24** | CBS domain/Bateman module  sensing cellular energy status, metal ion concentration, or ionic strength | (24, 25) | CBS domain  *Clostridium perfringes*  (PDB ID: 3L2B) | WP_011026007.1 | WP_003870521.1; WP_072969406.1; WP_003868321.1; WP_039929467.1; WP_012995611.1 | Cytoplasmic | Carbohydrate metabolism | putative |
| **25** | Polymerase III  replication of bacterial genome | (26, 27) | PolIII from *Thermus aquaticus*  (PDB ID: 2HPI) | WP_170270986.1; WP_132039922.1; WP_031313863.1 | WP_049685526.1;WP_012995610.1; WP_013150583.1; WP_028991897.1; WP_042833459.1; WP_072969405.1; WP_009052939.1; WP_006569387.1; WP_012269130.1; WP_003870520.1; WP_074666192.1; WP_004401580.1; WP_014063156.1 | Cytoplasmic | Replication | putative |
| **26** | P_II_-type signal-transducer  Regulation of both regulatory and catalytic proteins from nitrogen glutamine/glutamate metabolism | (28) |  | WP_011026005.1 | WP_012995609.1; WP_003868324.1; WP_028991898.1; WP_029688593.1; WP_014063155.1; WP_049685525.1 | Cytoplasmic | signal-transducing | putative |
| **27** | Aminopeptidase (PepT)  anaerobic regulated deblocking aminotripeptidase | (40) | PepT from *Salmonella typhimurium*  (PDB ID: 1FNO) | WP_022587067.1; WP_011025997.1; WP_170271716.1 | WP_049685514.1; WP_004401542.1; WP_039913318.1; WP_003871411.1; WP_072968240.1 | Cytoplasmic | proteasome | putative |
| **28** | Anaerobic *pep*T transcriptional activator *fnr* | (41) | PepT  *Bacillus anthracis*  (PDB ID: 3IFE) | AAM24999.1; WP_031313864.1; WP_043884268.1 | WP_004401536.1; WP_009052927.1; WP_003868335.1; WP_074665344.1; WP_072968242.1; WP_003871410.1; WP_013150567.1; WP_012995584.1 | Cytoplasmic | regulator | putative |
| **29** | methylenetetrahydrofolate reductase  (EC 1.5.1.20)  Methionine synthesis via methyl-THF | (44) |  | WP_022587070.1; WP_011025993.1  WP_170271719.1 | WP_012995581.1; WP_013150564.1  WP_028992614.1; WP_072968248.1  WP_006569949.1; WP_004400036.1  WP_003871154.1; WP_003868338.1; WP_074665348.1; WP_074592878.1 | Cytoplasmic | SAM, methionine, cysteine metabolism | putative |
| **30** | Methionine synthase  (EC 2.1.1.13) | (44) |  | WP_022587071.1; WP_011025992.1  WP_170271720.1; WP_009611154.1  WP_132040712.1 | WP_012269136.1; KUJ90444.1  WP_003868339.1; WP_013150563.1  WP_012995580.1; WP_028992615.1  WP_072968250.1; WP_003871153.1  WP_019907846.1; WP_014063140.1  WP_074592877.1; WP_004400037.1 | Cytoplasmic | SAM, methionine, cysteine metabolism | putative |
| **31** | tRNA(m^5^U_54_)  methyltransferase  (EC 2.1.1.190)  possible role in tRNA maturation | (36) | RNA methyltransferase  *S. pneumoniae*  (PDB ID: 5XJ1) | WP_011026001.1; WP_132040702.1; WP_170271819.1 | WP_012269132.1; WP_074666188.1; WP_004401568.1; WP_006569385.1; WP_003871319.1; WP_003868328.1; WP_014063153.1; WP_019907854.1; WP_072969401.1; WP_049685520.1; WP_028991900.1; WP_013150581.1; WP_012995605.1; | Cytoplasmic | RNA translation | putative |
| **32** | drug/Na^+^ MATE-like antiport  Multi-Antimicrobial Extrusion protein (MATE) a drug/Na+ antiport mechanism | (37) | MATE-like antiport *Aquifex aeolicus*  (PDB ID: 6FV6) | WP_011026000.1; WP_132040704.1; WP_170271820.1 | WP_006569382.1; WP_072969399.1; WP_014063150.1; WP_042833451.1; WP_012269133.1; WP_004401556.1; WP_009052935.1; WP_012995603.1; WP_003868330.1; WP_083768277.1; WP_081723115.1; WP_003871322.1; WP_049685517.1; | Membrane | Drug antiporter | putative |
|  | MsmX-like | (45, 46) | MsmX from *B. subtilis*  (PDB ID: 6YIR) | WP_011026113.1  MBE3578580.1  WP_132038984.1  KUK09439.1 | WP_003866589.1; WP_004400497.1  WP_006569486.1; WP_014063280.1  WP_003869434.1; WP_009052770.1  WP_012995743.1; WP_015311043.1 | Cytoplasmic | Carbohydrate transport | putative |
|  | phosphoglucomutase (Pgm) |  |  | WP_011025196.1 WP_170269961.1 WP_132039081.1 | WP_003866992.1; WP_012269468.1 | Cytoplasmic | Carbohydrate central metabolism | putative |

^a^ According to a bioinformatics pipeline described in the Methods section.

^b^ As predicted by CELLO v.2.5 analysis .

**REFERENCES**

1. Vemula H, Ayon NJ, Gutheil WG. 2015. Cytoplasmic peptidoglycan intermediate levels in *Staphylococcus aureus*. Biochimie 121:72–78.

2. Benson TE, Harris MS, Choi GH, Cialdella JI, Herberg JT, Martin JP, Baldwin ET. 2001. A Structural Variation for MurB : X-ray Crystal Structure of *Staphylococcus aureus* UDP-N-Acetylenolpyruvylglucosamine reductase (MurB). Biochemistry 40:2340–2350.

3. Aravind L, Koonin E V. 1998. Phosphoesterase domains associated with DNA polymerases of diverse origins. Nucleic Acids Research 26:3746–3752.

4. Geno KA, Hauser JR, Gupta K, Yother J. 2014. *Streptococcus pneumoniae* Phosphotyrosine Phosphatase CpsB and Availability. Journal of bacteriology 196:1992–2003.

5. Gonzalez GM, Durica-mitic S, Hardwick SW, Moncrieffe MC, Resch M, Neumann P, Ficner R, Boris G, Luisi BF. 2017. Structural insights into RapZ-mediated regulation of bacterial amino-sugar metabolism. Nucleic Acids Research 45:10845–10860.

6. Greening C, Ahmed FH, Mohamed AE, Lee BM, Pandey G, Warden AC, Scott C, Oakeshott JG, Taylor MC, Jackson J. 2016. Physiology, biochemistry, and applications of F420- and Fo-dependent redox reactions. Microbial and Molecular Biology Reviews 80:451–493.

7. Oyugi MA, Bashiri G, Baker EN, Johnson-Winters KL. 2016. Investigating the reaction mechanism of F420-dependent glucose-6-phosphate dehydrogenase from *Mycobacterium tuberculosis*: kinetic analysis of the wild-type and mutant enzymes. Biochemistry 55:5566–5577.

8. Tonozuka T, Sogawa A, Yamada M, Matsumoto N, Yoshida H, Kamitori S, Ichikawa K, Mizuno M, Nishikawa A, Sakano Y. 2007. Structural basis for cyclodextrin recognition by *Thermoactinomyces vulgaris* cyclo/maltodextrin‐binding protein. FEBS Journal 274:2109–2120.

9. Hulsmann A, Lurz R, Scheffel F, Schneider E. 2000. Maltose and maltodextrin transport in the thermoacidophilic gram-positive bacterium *Alicyclobacillus acidocaldarius* is mediated by a high-affinity transport system that includes a maltose binding protein tolerant to low pH. Journal of Bacteriology 182:6292–6301.

10. Pajatsch M, Gerhart M, Peist R, Horlacher R, Boos W, Böck A. 1998. The periplasmic cyclodextrin binding protein *cym*E from *Klebsiella oxytoca* and its role in maltodextrin and cyclodextrin transport. Journal of Bacteriology 180:2630–2635.

11. Hashimoto Y, Yamamoto T, Fujiwara S, Takagi M, Imanaka T. 2001. Extracellular synthesis, specific recognition, and intracellular degradation of cyclomaltodextrins by the hyperthermophilic archaeon *Thermococcus* sp. strain B1001. Journal of Bacteriology 183:5050–5057.

12. Hans M, Sievers J, Müller U, Bill E, Vorholt JA, Linder D, Buckel W. 1999. 2-Hydroxyglutaryl-CoA dehydratase from *Clostridium symbiosum*. European journal of biochemistry/FEBS 265:404–414.

13. Hofmeister AEM, Buckel W. 1992. (R)-Lactyl-CoA dehydratase from *Clostridium propionicum* stereochemistry of the dehydration of (R)-2-hydroxybutyryl-CoA to crotonyl-CoA. European journal of biochemistry/FEBS 552:547–552.

14. Muchová K, Chromiková Z, Valencíková R, Barák I. 2018. Interaction of the morphogenic protein RodZ with the *Bacillus subtilis* Min System. Frontiers in Microbiology 8:1–10.

15. Stamsas GA, Straume D, Winther AR, Kjos M, Frantzen CA, Havarstein LS. 2017. Identification of EloR (Spr1851) as a regulator of cell elongation in *Streptococcus pneumoniae*. Molecular Microbiology 105:954–967.

16. Podkovyrov SM, Zeikus JG. 1992. Structure of the gene encoding cyclomaltodextrinase from *Clostridium thermohydrosulfuricum* 39E and characterization of the enzyme purified from *Escherichia coli*. Journal of Bacteriology 174:5400–5405.

17. Ryding NJ, Hartley N, Findlay KC, Bruton C, KF C. 2000. WhiA, a protein of unknown function conserved among Gram-positive Bacteria, is essential for sporulation in *Streptomyces coelicolor* A3 (2). Journal of Bacteriology 182:5470–5478.

18. Kaiser BK, Clifton MC, Shen BW, Stoddard BL. 2009. The structure of a bacterial DUF199/WhiA protein: Domestication of an invasive endonuclease. Cell Structure 17:1368–1376.

19. Bohorquez LC, Surdova K, Jonker MJ, Hamoen LW. 2018. Chromosome segregation in *Bacillus subtilis*. Journal of Bacteriology 200:1–17.

20. Zhu M, Lu Y, Wang J, Li S, Wang X. 2015. Carbon catabolite repression and the related genes of *ccp*A, *pts*H and *hpr*K in *Thermoanaerobacterium aotearoense*. PLoS ONE 7:1–18.

21. Navdaeva V, Zurbriggen A, Waltersperger S, Schneider P, Oberholzer AE, Priska B, Christoph B, Grieder A, Baumann U, Erni B. 2011. Phosphoenolpyruvate:sugar phosphotransferase system from the hyperthermophilic *Thermoanaerobacter tengongensis*. Biochemistry 50:1184–1193.

22. Chunyan F, Feng GAO, Yiwei LIU, Guihua W, Hao P, Yanhe MA, Jinghua YAN, Fu GAOG. 2011. Crystal structure of histidine-containing phosphocarrier protein from *Thermoanaerobacter tengcongensis* MB4 and the implications for thermostability. Sci China Life Sci 54:513–519.

23. Oberholzer AE, Bumann M, Schneider P, Siebold C, Baumann U, Erni B. 2005. Crystal structure of the phosphoenolpyruvate-binding enzyme I-domain from the *Thermoanaerobacter tengcongensis* PEP:Sugar Phosphotransferase System (PTS). J Mol Biol 346:521–532.

24. Anashkin VA, Salminen A, Tuominen HK, Orlov VN, Lahti R, Baykov AA. 2015. Cystathionine beta Synthase (CBS) Domain-containing pyrophosphatase as a target for diadenosine polyphosphates in bacteria. The Journal of biological chemistry 290:27594–27603.

25. Baykov AA, Tuominen HK, Lahti R. 2011. The CBS Domain: A protein module with an emerging prominent role in regulation. ACS Chemical Biology 6:1156–1163.

26. Bailey S, Wing RA, Steitz TA. 2006. The structure of *T. aquaticus* DNA polymerase III is distinct from eukaryotic replicative DNA polymerases. Cell 126:893–904.

27. Timinskas K, Balvocluté M, Timiniskas A, Vencolvas C. 2014. Comprehensive analysis of DNA polymerase III a subunits and their homologs in bacterial genomes. Nucleic Acids Research 42:1393–1413.

28. Arcondeguy T, Jack R, Merrick M. 2001. P II Signal Transduction Proteins, Pivotal Players in Microbial Nitrogen Control. Microbiology and Molecular Biology Reviews 65:80–105.

29. Bapteste E, Moreira D, Philippe H. 2003. Rampant horizontal gene transfer and phospho-donor change in the evolution of the phosphofructokinase. Gene 318:185–191.

30. Zhou C-Q, Wang J-Q, Qian Z, Ma Y-H, Liu S-Q. 2006. Cloning, expression and characterization of the 6-phosphofructokinase from *Thermoanaerobacter tengcongensis*. Wei Sheng Wu Xue Bao 46:249–254.

31. Suzuki K, Ito S, Shimizu-ibuka A, Sakai H. 2008. Crystal Structure of Pyruvate Kinase from Geobacillus stearothermophilus. Journal of Biochemistry 312:305–312.

32. Zoraghi R, Worrall L, See RH, Strangman W, Popplewell WL, Gong H, Samaai T, Swayze RD, Kaur S, Vuckovic M, Finlay BB, Brunham RC, Mcmaster WR, Davies-coleman MT, Strynadka NC, Andersen RJ, Reiner NE. 2011. Methicillin-resistant *Staphylococcus aureus* (MRSA) pyruvate kinase as a target for bis-indole alkaloids with antibacterial. The Journal of biological chemistry 286:44716–44725.

33. Tillander V, Alexson SEH, Cohen DE. 2018. Deactivating Fatty Acids: Acyl-CoA Thioesterase-mediated control of lipid metabolism. Trends Endocrinol Metabolism 28:473–484.

34. Aleshin AE, Feng P, Honzatko RB, Reilly PJ. 2003. Crystal structure and evolution of a prokaryotic glucoamylase. J Mol Biol 2836:61–73.

35. Zheng Y, Xue Y, Zhang Y, Zhou C, Schwaneberg U, Ma Y. 2010. Cloning, expression, and characterization of a thermostable glucoamylase from *Thermoanaerobacter tengcongensis* MB4. Appl Microbiol Biotechnol 87:225–233.

36. Johansson MJO, Byström AS. 2002. Dual function of the tRNA (m5U54) methyltransferase in tRNA maturation. RNA 8:324–335.

37. Kusakizako T, Miyauchi H, Ishitani R, Nureki O. 2020. Structural biology of the multidrug and toxic compound extrusion superfamily transporters. BBA - Biomembranes 1862:183154.

38. Li J, Cai S, Luo Y, Dong X. 2011. Three feruloyl esterases in *Cellulosilyticum ruminicola* H1 act synergistically to hydrolyze esterified polysaccharides. Applied and environmental microbiology 77:6141–6147.

39. Abokitse K, Wu M, Bergeron H, Grosse S, Lau PCK. 2010. Thermostable feruloyl esterase for the bioproduction of ferulic acid from triticale bran. Applied microbiology and biotechnology 87:195–203.

40. Dutoit R, Brandt N, Legrain C, Bauvois C. 2012. Functional characterization of two M42 aminopeptidases erroneously annotated as cellulases 7.

41. Lombardo M, Lee AA, Knox TM, Miller CG. 1997. Regulation of the *Salmonella typhimurium* *pep*T gene by cyclic AMP receptor protein (CRP) and FNR acting at a hybrid CRP-FNR site. Journal of bacteriology 179:1909–1917.

42. Chou C-C, Sun Y, Meng M, Hsiao C-D. 2000. The crystal structure of phosphoglucose isomerase/autocrine motility factor/neuroleukin complexed with its carbohydrate phosphate inhibitors suggests its substrate/receptor recognition. The Journal of biological chemistry 275:23154–23160.

43. Chen S, Liu J, Pei H, Li J, Zhou J, Xiang H. 2007. Molecular investigation of a novel thermostable glucan phosphorylase from *Thermoanaerobacter tengcongensis*. Enzyme and Microbial Technology 41:390–396.

44. Rodionov DA, Vitreschak AG, Mironov AA, Gelfand MS. 2004. Comparative genomics of the methionine metabolism in Gram-positive bacteria: a variety of regulatory systems. Nucleic Acids Research 32:3340–3353.

45. Ferreira MJ, De Sá-Nogueira I. 2010. A multitask ATPase serving different ABC-type sugar importers in *Bacillus subtilis*. Journal of Bacteriology 192:5312–5318.

46. Leisico F, Godinho LM, Gonçalves IC, Silva SP, Carneiro B, Romão MJ, Santos-Silva T, de Sá-Nogueira I. 2020. Multitask ATPases (NBDs) of bacterial ABC importers type I and their interspecies exchangeability. Scientific Reports 10:1–17.
